# Comprehensive chemical characterization of the aerosol generated by a heated tobacco product by untargeted screening

**DOI:** 10.1007/s00216-020-02502-1

**Published:** 2020-02-18

**Authors:** Mark C. Bentley, Martin Almstetter, Daniel Arndt, Arno Knorr, Elyette Martin, Pavel Pospisil, Serge Maeder

**Affiliations:** Philip Morris International Research and Development, Philip Morris Products S.A, Quai Jeanrenaud 3, 2000 Neuchâtel, Switzerland

**Keywords:** Chemical characterization, Untargeted screening, Heated tobacco product, Aerosol, GC×GC-TOFMS, LC-HRAM-MS

## Abstract

A suite of untargeted methods has been applied for the characterization of aerosol from the Tobacco Heating System 2.2 (THS2.2), a heated tobacco product developed by Philip Morris Products S.A. and commercialized under the brand name *IQOS*®. A total of 529 chemical constituents, excluding water, glycerin, and nicotine, were present in the mainstream aerosol of THS2.2, generated by following the Health Canada intense smoking regimen, at concentrations ≥ 100 ng/item. The majority were present in the particulate phase (*n* = 402), representing more than 80% of the total mass determined by untargeted screening; a proportion were present in both particulate and gas-vapor phases (39 compounds). The identities for 80% of all chemical constituents (representing > 96% of the total determined mass) were confirmed by the use of authentic analytical reference materials. Despite the uncertainties that are recognized to be associated with aerosol-based untargeted approaches, the reported data remain indicative that the uncharacterized fraction of TPM generated by THS2.2 has been evaluated to the fullest practicable extent. To the best of our knowledge, this work represents the most comprehensive chemical characterization of a heated tobacco aerosol to date.

**Electronic supplementary material:**

The online version of this article (10.1007/s00216-020-02502-1) contains supplementary material, which is available to authorized users.

**Figure:**
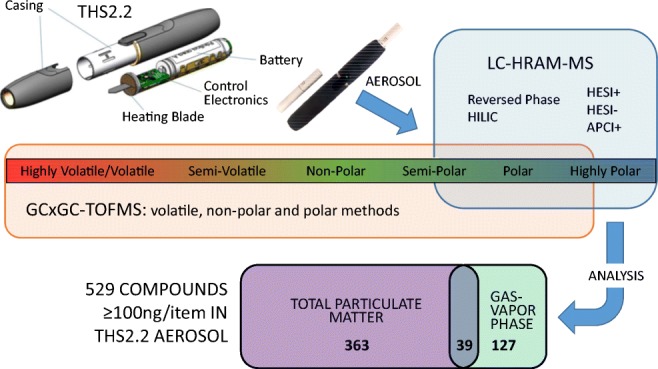
Graphical abstract

## Introduction

It is well recognized that cigarette smoking is one of the leading causes of preventable death worldwide [1]. For decades, harm reduction efforts have been focused on preventing smoking initiation and promoting smoking cessation. However, the majority of smoking-related diseases are unrelated to nicotine itself [2] but associated with other toxicants produced from the combustion of tobacco and inhaled by the smoker. In light of this fact, complete cessation of all tobacco and nicotine use should be the ultimate goal for preventing harm from smoking. However, given the number of adult smokers who will continue to smoke cigarettes, estimated at more than 1 billion by 2025, switching to sources of nicotine that do not involve tobacco combustion is seen as an alternative approach to harm reduction, without the need to overcome any addiction to nicotine [3, 4]. To this end, a number of companies, including Philip Morris Products S.A. (PMP), are developing heated tobacco products, which are designed to significantly reduce or eliminate the levels of toxicants found in their aerosol while attempting to preserve the taste, sensory experience, nicotine delivery profile, and ritual characteristics of cigarettes. Such products are intended to meet the requirements of the US Family Smoking Prevention and Tobacco Control Act (FSPTCA, 2009) [5] in order to be defined as modified risk tobacco products (MRTP), thereby allowing their sale or distribution for reducing the harm or risk of tobacco-related diseases associated with commercially marketed tobacco products.

Cigarette smoke is reported to contain more than 6000 chemical constituents [6], which are formed during distillation, pyrolysis, and combustion reactions when tobacco is burned [7]. Various scientific and regulatory bodies have also acknowledged the presence of more than 100 harmful and potentially harmful constituents (HPHC) in tobacco and cigarette smoke [8–10]. Heated tobacco products are designed to heat tobacco to temperatures below that required to initiate combustion (ca. 400 °C), where an aerosol is generated by the process of evaporation, distillation, and low-temperature pyrolysis of the tobacco substrate, which would include processing agents used during manufacture and any flavor additives, resulting in the production of reduced levels of HPHCs. The Tobacco Heating System 2.2 (THS2.2, commercialized under the brand name *IQOS*®) is a heated tobacco product developed by PMP, for which the absence of combustion has been confirmed [11]. In order to provide evidence for MRTP classification, PMP uses a structured scientific assessment program that covers both non-clinical and clinical evaluation, which has been applied for assessment of THS2.2 [12]. Part of the non-clinical assessment for THS2.2 involved the targeted measurement of priority toxicants listed by the World Health Organization [13], Health Canada [14], and the US Food and Drug Administration (FDA; reduced panel of 18 constituents) [15], all of which are present in the list of 54 HPHCs routinely measured at PMP for the development of heated tobacco products. Significant reductions have been reported in the levels of all these priority toxicants in the mainstream aerosol of THS2.2 relative to the mainstream smoke of the 3R4F reference cigarette [16], with the majority of HPHC concentrations being comparatively reduced by more than 90% [17].

However, despite these reductions in mainstream aerosol concentrations, and an associated reduction for in vitro toxicity compared with that of 3R4F mainstream smoke [17], there is lingering skepticism within the scientific community as to whether the priority toxicants identified for regulation of cigarettes are directly applicable for assessment of heated tobacco products. Because lower temperatures are used for aerosol formation in such products, where combustion does not occur, the question always arises as to whether a different palate of toxicologically relevant compounds is generated. To generalize, the open question is always “What exactly is in the aerosol?” To answer this question, in this publication, we present the results for the characterization of THS2.2 aerosol using a suite of untargeted analytical methods. To the best of our knowledge, this is the most comprehensive chemical characterization of a heated tobacco aerosol reported to date.

## Materials and methods

### Approach

**Fig. 1:**
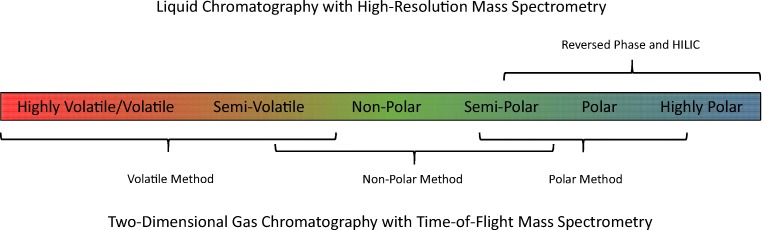
Illustration of the suite of untargeted methods used to cover the anticipated chemical space for tobacco smoke and aerosol, indicating approximate overlaps between the methods (HILIC, hydrophilic interaction liquid chromatography)

In contrast to quantitative analysis, where chemical constituents (analytes) of interest are targeted to the exclusion of all others, an untargeted approach considers indiscriminate determination of all analytes relevant to a specific chemical space. To this end, a portfolio of methods has been developed that aims to deliver maximum coverage for the chemical space related to tobacco-derived aerosols (Fig. 1). These methods are based on comprehensive two-dimensional gas chromatography with time-of-flight mass spectrometry (GC×GC-TOFMS) [18] and liquid chromatography with high-resolution accurate mass spectrometry (LC-HRAM-MS) [19]. They are semi-quantitative in nature, whereby the concentrations of a large number of compounds are estimated versus a limited number of stable isotope-labeled reference standards of known concentrations, using an internal standardization approach. For constituents amenable to gas chromatographic analysis, quantitative values derived from analysis of recognized HPHCs by using targeted methods were compared with corresponding semi-quantified values from untargeted analysis. This demonstrated that, for analytes that were measureable by both techniques, the untargeted approach using GC×GC-TOFMS was able to semi-quantify within a ± 4-fold deviation from the true concentration [18]. Available liquid chromatography–based targeted methods were insufficient to make a similar assessment for LC-HRAM-MS. Therefore, semi-quantification for this approach, as described here, is sufficient to estimate concentrations within a correct order of magnitude. Because the chemical diversity of constituents within a tobacco-derived aerosol is so large, a number of overlapping methods that use both gas and liquid chromatography are required to ensure maximal analytical coverage. However, it should be recognized that not all compounds present will be detected, and, despite the comprehensive nature of this analytical approach, a very small proportion of constituents may be overlooked. For example, mass spectrometers are not able to detect metals in their elemental state or molecules that cannot be electrically charged to create ions.

An untargeted assessment approach is a time-consuming process, especially in the post-analysis data treatment stage, because each chromatographic peak for each individual chemical constituent needs to be evaluated in order to propose a chemical name and provide a semi-quantified concentration. In order to streamline this process, compounds were identified by using software platforms that automated the iterative steps required for accurately identifying each chemical constituent. For GC×GC-TOF methods, an in-house platform was used, which interrogated both commercially available and custom-built [20] mass spectral databases for spectral similarity and compared a number of predicted and measured parameters, such as retention index or relative/absolute retention time in both chromatographic dimensions in order to improve the confidence of identification [21]. For LC-HRAM-MS, a similar workflow was implemented by using the Progenesis QI™ software (Nonlinear Dynamics, Newcastle upon Tyne, UK), which also incorporated a modified MetFrag in silico fragmentation algorithm [22, 23] to enable comparison of experimental and predicted first-order fragmentation spectra.

These methods were applied for analysis of the particulate and gas-vapor phases of aerosols generated by a heated tobacco product and cigarette smoke, represented by THS2.2 and 3R4F, respectively. The aim was to comprehensively characterize the composition of THS2.2 aerosol and provide comparative concentration data for those constituents also confirmed to be present in cigarette smoke.

### Aerosol generation and analysis

**Fig. 2:**
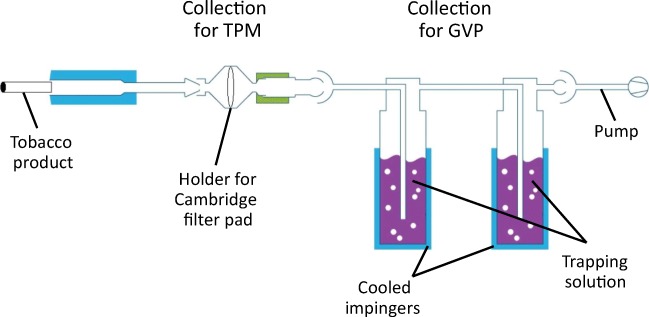
General schematic for smoke/aerosol trapping (TPM, total particulate matter; GVP, gas-vapor phase)

Because data from the different analytical methods were required to be combined, it was essential to ensure comparability between trapping approaches. To this end, a harmonized approach was adopted that employed separate trapping of the particulate and gas-vapor phases (which together comprise whole aerosol or smoke for THS2.2 or 3R4F, respectively), thereby enabling individual chemical characterization of these two distinct phases. Aerosol from THS2.2 and smoke from the reference cigarette 3R4F were generated by using a linear smoking machine in accordance with the Health Canada intense (HCI) smoking regimen [24]. The particulate phase, commonly referred to as total particulate matter (TPM), was collected by passing the smoke/aerosol through a Cambridge (glass fiber) filter pad (CFP), and the gas-vapor phase (GVP) passing through the CFP was trapped by using two micro-impingers in series, which contained solvents maintained at sub-ambient temperatures (Fig. 2). For GC×GC-TOFMS analysis, internal standards for semi-quantification were present within the solvents used to extract the CFPs, and within the solvents used to trap the GVP. For LC-HRAM-MS, internal standards were added to samples after the aerosol collection process, but before chromatographic analysis. 3R4F samples were collected as an accumulation of smoke from up to three cigarettes, and for THS2.2, an accumulation of aerosol from up to five heated tobacco “sticks” was collected for analysis. The number of accumulations collected was optimized to match the requirements for individual analytical methods, and replicate sample collection was performed for each required measurement (at least triplicate). The samples were subsequently analyzed by GC×GC-TOFMS [18] and LC-HRAM-MS [19].

**Fig. 3:**
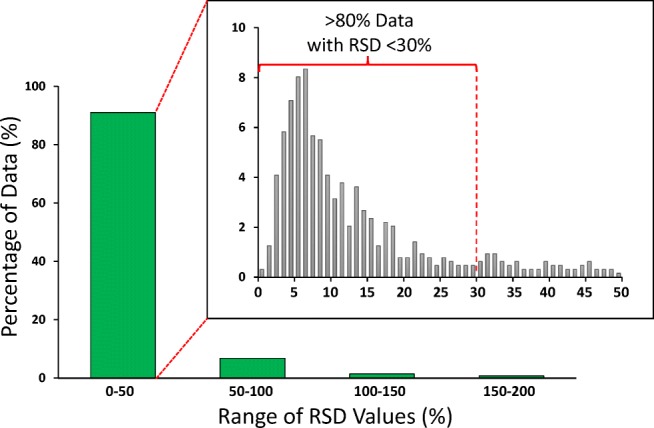
Overall distribution of RSD values observed for non-targeted analysis replicates (RSD, relative standard deviation)

For GC×GC-TOFMS, the aerosol or smoke collections (×3 for polar and non-polar methods, ×4 for volatile method) were each injected once for chromatographic analysis. Replicate injections for GC×GC-TOFMS analysis were not performed due to the highly complex nature of the sample extracts, particularly those for the 3R4F reference cigarette, which reduced the effective lifetime of the chromatographic columns used and limited the number of injections that could be made per analytical run. For LC-HRAM-MS, the smoke or aerosol collections (×3) for each method were injected for chromatographic analysis 5 times. The values reported for all methods were the overall mean values for aerosol/smoke collections, and any injection replicates, without the exclusion of any data. Figure 3 presents a graphical representation for the overall variability of the analytical data generated, and shows that more than 80% of the mean values reported had calculated relative standard deviation (RSD) values of less than 30%.

For reporting purposes, a threshold of 100 ng/item (item = cigarette or heated tobacco “stick”) was employed, which was selected based upon the method performance assessment executed for the GC×GC-TOFMS workflow. During this method evaluation process, it was observed that the number of compounds identified to be present in the smoke of the 3R4F reference cigarette increased exponentially as their concentration decreased. By extrapolating this exponential trend and comparing the anticipated number versus the actual number of compounds identified, a point was reached at a concentration of 100 ng/item, below which the actual number of compounds identified was consistently lower than expected, which was interpreted as being the lower limit of the working range for the methodology [18]. While the number of compounds increased as their concentrations decreased, the mass contribution relative to the total chemical space identified declined logarithmically and the accumulated total mass for compounds below 100 ng/item for 3R4F was estimated to be less than 1% of the total mass amenable by GC×GC-TOFMS analysis [18]. A formal performance assessment specific for the LC-HRAM-MS methods has not yet been performed; however, the characteristics were assumed to be broadly comparable, and a 100 ng/item threshold was applied to data generated by both GC×GC-TOFMS and LC-HRAM-MS platforms, which served to optimize the proportion of substances identified relative to a practicable amount of effort applied for their identification. Detailed methodological information for sample collection and analysis is presented in the Electronic Supplementary Material (ESM).

## Results

### Whole aerosol (TPM and GVP)

Water, nicotine, and glycerin, being highly abundant in THS2.2 aerosol, were determined by using separate quantitative methods and were not considered as part of this untargeted assessment. Excluding these constituents, a total of 529 compounds with concentrations ≥ 100 ng/item were identified as being present in THS2.2 aerosol by untargeted screening. By applying this nominal reporting threshold of ≥ 100 ng/item, it was estimated that close to 100% of the total aerosol mass determined by untargeted screening was evaluated [18]. Table 1 presents information on the 100 most abundant compounds present in THS 2.2 aerosol as well as their corresponding concentrations in the smoke of the reference cigarette, 3R4F. A full list of the compounds identified is presented in the ESM.

**Table 1:** Top 100 most abundant chemical constituents present in THS2.2 aerosol (≥ 100 ng/item) and their corresponding concentrations in the smoke of the 3R4F reference cigarette

Compound name	CAS number	Identification confidence	Aerosol^a^ fraction	Conc. in THS2.2 (μg/item)	Conc. in 3R4F (μg/item)
1-Hydroxy-2-propanone/1,2-propenediol^b^	116-09-6/7333-03-1	Confirmed	TPM	1135	502
Acetic acid	64-19-7	Confirmed	TPM	994^c^	2659
Propylene glycol	57-55-6	Confirmed	TPM	643	89.6
1-Monoacetin	106-61-6	Confirmed	TPM	409	434
Acetaldehyde	75-07-0	Confirmed	TPM/GVP	313	1253
Methanol	67-56-1	Confirmed	TPM/GVP	211	361
Solanesol	13190-97-1	Confirmed	TPM	179	3382
Isobutyraldehyde	78-84-2	Confirmed	GVP	116	259
Triacetin	102-76-1	Confirmed	TPM	112	194
Palmitic acid	57-10-3	Confirmed	TPM	105	266
3-(2-Hydroxymethoxy)-propane-1,2-diol	14641-24-8	Confirmed	TPM	100	267
Cembranoid degradation products (18 compounds)^d^	–	Confirmed	TPM	93.2	193
Isovaleraldehyde	590-86-3	Confirmed	TPM/GVP	88.7	245
13,14-Dihydroretinol	115797-14-3	Confirmed	TPM	79.1	152
Linolenic acid	463-40-1	Confirmed	TPM	57.9	157
Propanal	123-38-6	Confirmed	GVP	57.4	386
2-Methylbutyraldehyde	96-17-3	Confirmed	GVP	54.7	179
Propanoic acid	79-09-4	Confirmed	TPM	53.2	141
3-Pyridinol	109-00-2	Confirmed	TPM	52.8	218
β-Nicotyrine	487-19-4	Confirmed	TPM	52.4	100
Pyranone	28564-83-2	Confirmed	TPM	51.4	44.5
Oleic acid	112-80-1	Confirmed	TPM	50.2	107
Furfural	98-01-1	Confirmed	TPM/GVP	47.4	38.3
2-Monoacetin	100-78-7	Confirmed	TPM	46.8	30.0
Linoleic acid	60-33-3	Confirmed	TPM	43.0	123
2-Furanmethanol	98-00-0	Confirmed	TPM/GVP	37.5	9.47
Acetone	67-64-1	Confirmed	GVP	34.7	268
2,3-Butanedione	431-03-8	Confirmed	TPM/GVP	34.0	127
Anhydro sugar derivative	–	High	TPM	30.8	43.1
Octadecanoic acid	57-11-4	Confirmed	TPM	29.4	72.7
2-Methylfuran	534-22-5	Confirmed	GVP	28.2	175
Furan	110-00-9	Confirmed	GVP	24.3	214
Neophytadiene	504-96-1	Confirmed	TPM	23.8	43.0
1-Linolenoylglycerol	18465-99-1	Confirmed	TPM	23.5	42.8
5-Hydroxymethylfurfural	67-47-0	Confirmed	TPM	23.0	82.1
α-Levantenolide	30987-48-5	Medium	TPM	22.8	74.8
2-Methyl-2-propenal	78-85-3	Confirmed	TPM/GVP	22.0	115
Pentadecanoic acid	1002-84-2	Confirmed	TPM	18.8	32.7
3-Chloro-1,2-propanediol	96-24-2	Confirmed	TPM	16.1	8.21
4,6-Dihydroxy-20-nor-2,7-cembradien-12-one	119613-98-8^e^	High	TPM	14.6	17.8
3-Methylpentanoic acid	105-43-1	Confirmed	TPM	14.5	12.8
5-Methylfurfural	620-02-0	Confirmed	TPM/GVP	14.2	5.25
1H-Pyrrole	109-97-7	Confirmed	TPM/GVP	14.0	24.8
Phytoene	540-04-5	Medium	TPM	13.8	247
Pyridine	110-86-1	Confirmed	TPM/GVP	13.7	68.4
6,10,14,18,22,26-Hexamethyl-5,9,13,17,21,25-heptacosahexaen-2-one	32304-17-9^e^	Medium	TPM	13.2	75.3
Butanoic acid	107-92-6	Confirmed	TPM	12.7	22.4
1-Acetyloxy-2-propanone	592-20-1	Confirmed	TPM/GVP	12.2	9.23
N-Octanoylnornicotine	38854-10-3	Confirmed	TPM	12.1	100
5,6-Dihydropyridin-2(1H)-one	6052-73-9	Confirmed	TPM	11.8	38.8
Methanethiol	74-93-1	Confirmed	GVP	11.7	22.9
Chloromethane	74-87-3	Confirmed	GVP	11.1	32.1
Heptacosane	593-49-7	Confirmed	TPM	10.2	8.41
α-Tocopherolquinone	7559-04-8	Confirmed	TPM	10.0	40.5
2-Butanone	78-93-3	Confirmed	GVP	10.0	128
3-Hydroxy-2-butanone	513-86-0	Confirmed	TPM/GVP	9.43	11.2
Arachidic acid	506-30-9	Confirmed	TPM	8.91	35.8
α-Cembratriene-diol	57605-80-8^e^	High	TPM	8.49	0.393
(9*Z*,12*Z*)-18-Hydroxy-9,12-octadecadienoic acid	4546-59-2	High	TPM	8.47	21.9
2-Cyclopentene-1,4-dione	930-60-9	Confirmed	TPM/GVP	8.40	2.01
2-Cyclopenten-1-one	930-30-3	Confirmed	TPM/GVP	8.20	46.9
2H-Pyran-2-one, tetrahydro-5-hydroxy	33691-73-5	Confirmed	TPM	8.16	4.13
2-Furancarboxylic acid, 3-methyl	4412-96-8	Confirmed	TPM	8.06	18.9
trans-Crotonaldehyde	123-73-9	Confirmed	TPM/GVP	7.87	210
8,11-Epoxy-2,6,12-cembratrien-4-ol	75281-94-6^e^	Medium	TPM	7.79	19.6
Butanal	123-72-8	Confirmed	GVP	7.79	114
trans-Solanone	54868-48-3	Confirmed	TPM/GVP	7.75	12.7
Palmitoleic acid	373-49-9	Confirmed	TPM	7.46	22.0
Isoraimonol	82458-63-7	High	TPM	7.42	11.4
Scopoletin	92-61-5	Confirmed	TPM	7.21	42.3
Anatabine	581-49-7	Confirmed	TPM	7.15	11.8
Behenic acid	112-85-6	Confirmed	TPM	6.57	40.1
2,3-Pentanedione	600-14-6	Confirmed	GVP	6.43	17.0
Hexadecanoic acid, ethyl ester	628-97-7	Confirmed	TPM	6.43	< 0.100
2,5-Dimethylfuran	625-86-5	Confirmed	GVP	6.38	156
Dimethyl disulfide	624-92-0	Confirmed	GVP	6.34	74.1
2-Methyl-3-pyridinol	1121-25-1	Confirmed	TPM	6.23	41.0
5-Oxo-1-tetradecyl-3-pyrrolidinecarboxylic acid	10054-22-5	Medium	TPM	6.16	10.8
α-Tocopherol	10191-41-0	Confirmed	TPM	5.80	25.4
1,2-Benzenediol	120-80-9	Confirmed	TPM	5.73	56.5
Hydroquinone	123-31-9	Confirmed	TPM	5.71	80.6
2-Hydroxy-3-oxo-butanal	473-80-3	High	TPM/GVP	5.60	6.58
Andrograpanin	82209-74-3	Confirmed	TPM	5.57	61.7
N-Cyclohexylnicotinamide	10354-56-0	Medium	TPM	5.56	48.3
Lignoceric acid	557-59-5	Confirmed	TPM	5.47	30.1
2(5H)-Furanone	497-23-4	Confirmed	TPM	5.45	2.13
2-Methylbutanoic acid	116-53-0	Confirmed	TPM	5.28	8.08
Isoprene	78-79-5	Confirmed	GVP	5.24	49.1
Acrolein	107-02-8	Confirmed	GVP	5.20	463
3-Methylbutanoic acid	503-74-2	Confirmed	TPM	5.13	9.98
6-Methyl-3-pyridinol	1121-78-4	Confirmed	TPM	5.10	21.3
1,4,7,10-Cyclotetradecatetraene, 1,7,11-trimethyl-4(1-methylethenyl)	101159-07-3	High	TPM	4.93	11.9
Butyrolactone	96-48-0	Confirmed	TPM	4.80	1.08
Myristic acid	544-63-8	Confirmed	TPM	4.62	18.7
Stearidonic acid	20290-75-9	Confirmed	TPM	4.56	21.3
Hentriacontane	630-04-6	Confirmed	TPM	4.54	20.4
Benzene	71-43-2	Confirmed	GVP	4.41	106
Acetamide	60-35-5	Confirmed	TPM	4.30	38.7
3-Methylpalmitic acid	42172-35-0	Medium	TPM	4.27	11.7

^a^*TPM*, total particulate matter; *GVP*, gas-vapor phase

^b^Semi-quantified concentration represents the sum value for two tautomers, which interconvert inconsistently during analysis

^c^Concentration determined quantitatively

^d^Degradation experiments with suspected precursors were performed, which confirmed the compound class proposal

^e^CAS number corresponds to one of the isomeric forms for this compound

Three confidence categories were used for structural identification proposals (high, medium, and not identified) based on the combined scores calculated by using mass spectral similarity as well as the comparison of additional predicted and experimental parameters [18, 19]. Once structural proposals were confirmed by comparative analysis using authentic analytical reference standards, the status “confirmed” was assigned to the compounds. During the evaluation of the GC×GC-TOFMS methodology, which was focused upon the smoke of the 3R4F reference cigarette, it was observed that there were higher numbers of reliably identified compounds compared with unidentified compounds where their abundance exceeded 100 ng/item. For compounds of lower abundance, the number of unidentified constituents increased dramatically in comparison with those with reliable proposals, which was most likely attributable to lower spectral quality affecting the identification process. However, other factors may also have contributed, such as the increased presence of less-common structures at lower concentrations that were absent from commercially available mass spectral databases. Focusing on the compounds that were confirmed to be present by reference standard, and also present in commercial mass spectral libraries, the success rate for the GC×GC-TOFMS workflow to propose the true structure as the top “hit” for unknowns classified with “high confidence” (220 compounds) was greater than 90%. For “medium confidence” proposals (17 compounds), this success rate reduced to ca. 75% [18]. For LC-HRAM-MS, the success rate for the workflow to propose the true structure as the top “hit” for unknowns classified with “high confidence” in this investigation was estimated to be lower at 62%. However, this success rate was achieved by inclusion of an in-house experimental mass spectral library in the compound identification workflow. When relying solely on comparisons with mass spectra available in commercially available libraries, this success rate reduced to 14%, primarily due to the fact that the majority of spectra within these libraries are generated using gas chromatography with high energy electron ionization, which are generally not representative of the spectra generated by LC-HRAM-MS ionization processes.

**Fig. 4:**
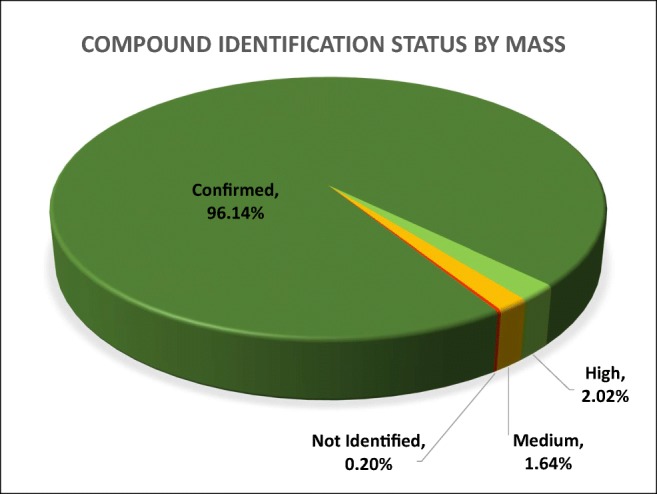
Distribution of compound identification status by mass

In this investigation, ca. 67% of the compounds identified by LC-HRAM-MS were confirmed by reference standard, versus ca. 87% for GC×GC-TOFMS, reflective of the lower success rate for compound identification using the LC-HRAM-MS workflow. Overall, 80% of the total number of structural proposals made was confirmed by the use of reference standards. In terms of the total mass characterized, more than 96% was confirmed by reference standards, with only 0.2% of the total mass being assigned as “not identified” (Fig. 4). Compounds identified with either “high” or “medium” confidence are yet to be confirmed by a reference standard, primarily owing to their lack of commercial availability.

**Fig. 5:**
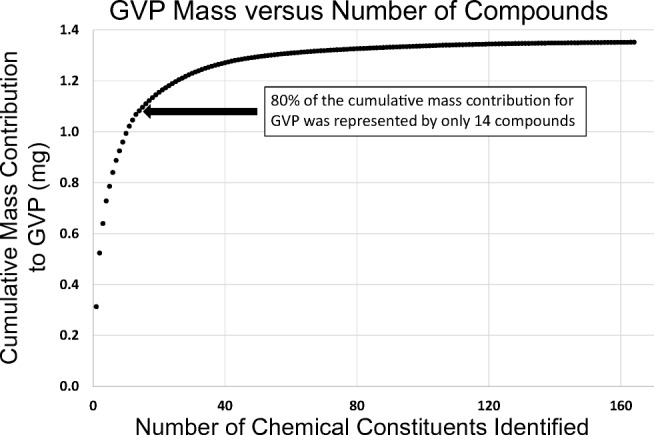
Cumulative mass of individual chemical constituents contributing to THS2.2 GVP, ranked in order from the highest (left) to lowest (right) individual mass contribution (GVP, gas-vapor phase)

Of the 529 compounds, 363 (68.6%) and 127 (24.0%) were exclusively found in the particulate and gas-vapor phases, respectively, and 39 (7.4%) compounds were partitioned between both particulate and gas-vapor phases. The particulate phase represented 81.3% of the total mass determined by untargeted screening. All 529 compounds were also determined to be present in the smoke of the 3R4F reference cigarette, with only a minority of the compounds being present in THS2.2 aerosol at concentrations exceeding those measured in the smoke of the 3R4F reference cigarette. The GVP, which represented just under 20% of the total mass determined by untargeted screening, was represented by 166 compounds. More than 80% of the determined mass for the GVP present in THS2.2 aerosol was contributed by the 14 most abundant chemical constituents, with more than 50% represented by just four constituents (see Fig. 5). It was not possible to compare the analytically determined mass contribution of the gas-vapor phase with any gravimetrically determined value, since it was not feasible to measure the mass of the gas-vapor phase generated using the trapping system, as described. Accordingly, no mass-based estimate for the achieved chemical coverage could be made. The major compound classes contributing to the GVP fraction were aldehydes, ketones, alcohols, and furanic compounds.

### Nicotine-free dry particulate matter

Cigarette smoke collection by using a CFP is routinely performed to determine nicotine, water, and nicotine-free dry particulate matter (NFDPM) (sometimes referred to as “tar”) concentrations by following a defined smoking regimen such as the HCI regimen [24]. The total mass of collected material (TPM) is obtained by calculating the weight difference of the CFP before and after smoke/aerosol collection. Water and nicotine are extracted from the CFP and quantified. Their masses are then subtracted from the TPM value to obtain the value for NFDPM (NFDPM = TPM − [water + nicotine]). However, it should be recognized that, although it is possible to formally calculate NFDPM values for both heated tobacco products and cigarettes, a direct comparison would be misleading because of the markedly higher proportion of humectants and lower levels of toxicants in the NFDPM of heated tobacco products than in cigarettes [25]. In addition, for THS2.2 aerosol, which is primarily composed of water, the standard methodology applied for cigarettes (ISO 4387:2000) [26] was not adequate to fully recover the increased amount of water retained by the CFP, which led to overestimation of NFDPM. An improved method was developed and used internally within PMP for accurately determining TPM delivery and calculating the amount of NFDPM collected [27]. The amount of glycerin collected was also quantitatively determined, since it is a key component of THS2.2 aerosol and is also used in cigarettes as a tobacco humectant.

**Fig. 6:**
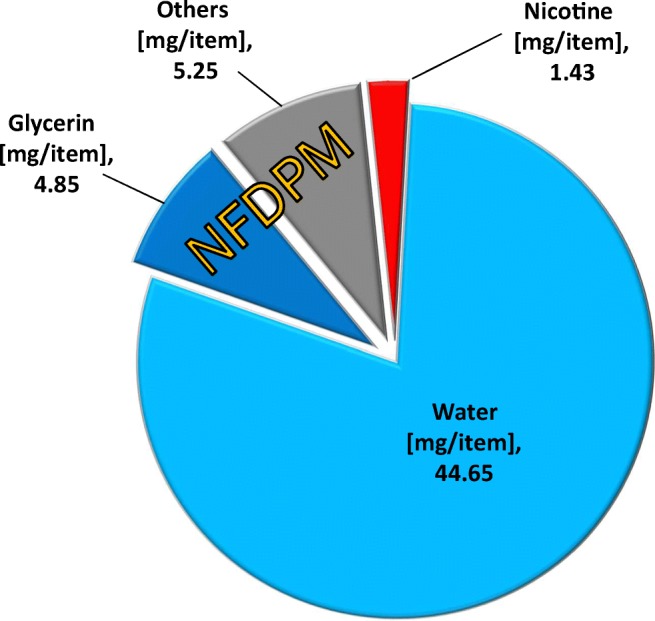
Gross composition of TPM from THS2.2 aerosol generated by using the Health Canada intense smoking regimen (TPM, total particulate matter; NFDPM, nicotine-free dry particulate matter)

Figure 6 shows the relative quantities of water, nicotine, and glycerin determined to be present in THS2.2 aerosol by using this improved methodology; here, NFDPM is represented as the sum of masses attributable to glycerin and “others.” The total mass of TPM delivered by THS2.2 aerosol was 56.18 mg/item (determined gravimetrically), with 5.25 mg of the NFDPM portion indicated as being of unknown composition or “others” (i.e., TPM − [water + glycerin + nicotine]). It should be noted that these measurements were performed independently of the smoke/aerosol collections used for untargeted screening, since the extraction method for measuring water, nicotine, and glycerin was not compatible for subsequent untargeted evaluation. Combining the uncertainties for each of the measures required to determine the mass of NFDPM with unknown composition (i.e., TPM, water, nicotine, and glycerin), a 95% confidence interval value of 0.82 should be considered alongside the estimated 5.25 mg amount.

**Fig. 7:**
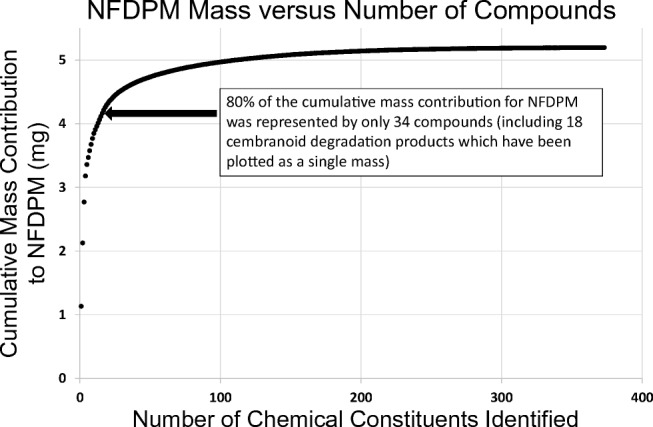
Cumulative mass of individual chemical constituents contributing to THS2.2 NFDPM (excluding glycerin) ranked in order from the highest (left) to lowest (right) individual mass contribution (NFDPM, nicotine-free dry particulate matter)

The total mass of NFDPM excluding glycerin (NFDPM-G) estimated by untargeted screening in this investigation was 5.20 mg, with both positive and negative deviations from true concentration, across the full range of compounds identified, assumed to be equivalent. Based upon estimated values, this represented 99% of this previously unknown portion. A total of 402 compounds with concentrations ≥ 100 ng/item were identified as being present in NFDPM-G. Approximately 97.9% (5.09 mg) of NFDPM-G estimated by untargeted screening comprised compounds that were either confirmed by using reference standards (95.4%) or identified with a high degree of confidence (2.5%). More than 80% of the estimated mass for NFDPM-G delivered by THS2.2 aerosol was represented by 34 chemical constituents, with over 50% represented by just the four most abundant constituents (see Fig. 7). Although the uncertainties associated with the experimental measurements made during the course of this investigation should be considered, these data demonstrate the most comprehensive characterization of TPM generated by THS2.2 that is technically achievable.

## Discussion

In order to maximize coverage of the chemical space related to heated tobacco aerosol and cigarette smoke, a suite of overlapping analytical methods was used, which was designed to cover the broadest possible range of chemical classes. Since the chemical complexity of a smoke or aerosol matrix is extremely high, sample collection and preparation processes were designed to be quick and simple, in order to minimize the occurrence of methodological artifacts. Of specific note was the approach taken to avoid the use of derivatization reagents for the analysis of polar compounds by GC×GC-TOFMS, where direct injection of aqueous samples onto the chromatographic system was performed. Thereby difficulties associated with the identification of compounds from their corresponding derivatives, due to the absence of corresponding spectra in commercially available databases, and further complicated by the fact that some compounds may derivatize in more than one position, were avoided. Although these methodologies were specifically developed to optimize analytical coverage, it is acknowledged that there will be some compromise associated with the application of non-specific sample collection and preparation processes, and that some classes of chemicals will be less amenable for analysis than others. However, a range of different solvents suited to the different (overlapping) methods have been used in order to minimize any shortfall, and only a small minority of constituents are considered to have been overlooked. For example, highly reactive compounds such as unstable radicals, which require specific analytical techniques for stabilization (or derivatization) prior to analysis, will be very short lived within the collection system. Formaldehyde, a highly reactive carbonyl known to be present in both cigarette smoke and heated tobacco aerosol, was also not detected. Mass spectrometric detection also has some limitations, with an inability to detect compounds below the pre-defined scan range (such as carbon monoxide), metals, inorganic acids, ammonia, and compounds that do not easily ionize. There is also an upper mass range limitation of ca. 800 Daltons for the detection of chemical constituents; however, this was not expected to have had a significant impact since compounds with a high molecular mass were not expected to have transferred substantially into the aerosol of a tobacco heating system that relies on volatility for transfer.

Excluding water, glycerin, and nicotine, 529 chemical constituents were identified as being present in THS2.2 aerosol at concentrations ≥ 100 ng/item. Of these compounds, 80% (representing > 96% of the total mass characterized by untargeted analysis) were confirmed by using authentic reference standard materials. The majority of compounds were present in the particulate phase (402, of which 39 were present in both gas-vapor and particulate phases), representing 81.3% of the total mass determined by untargeted screening. Combining the estimated masses for confirmed compounds and compounds identified with a high degree of confidence, while acknowledging the uncertainties associated with experimental measurements, it is reasonable to conclude that the most comprehensive characterization of TPM generated by THS2.2 that is technically achievable has been accomplished.

Mainstream smoke of the 3R4F reference cigarette was demonstrated to contain all 529 compounds that were present in THS2.2 aerosol (at concentrations ≥ 100 ng/item), and only a minority of compounds in THS2.2 aerosol were present at concentrations exceeding those measured in the smoke of the 3R4F reference cigarette. As such, one may broadly consider the composition of THS2.2 aerosol to be a subset of cigarette smoke, thereby rendering moot any assumption that a completely different palate of toxicologically relevant compounds will be present in the aerosol of heated tobacco products. Of course it is recognized that, with the application of a reporting threshold set at 100 ng/item, the presence of any compounds representing a toxicological concern at concentrations below this threshold would be overlooked. However, in the context of “new hazard” identification, PMP also uses the methods reported in this publication for a differential screening approach, which does not apply a concentration reporting threshold. A previous study, submitted to the FDA within the context of PMP’s MRTP application, was performed using this differential screening approach and the constituents that were found to be significantly higher in THS2.2 aerosol compared with 3R4F smoke, including three compounds that were unique to THS2.2 aerosol (cis-sesquisabinene hydrate, 61 ng/item; ethyl dodecanoate, 23 ng/item; and benzenemethanol, 4-hydroxy, 11 ng/item), were submitted for toxicological evaluation [28]. These three compounds unique to THS2.2 aerosol were all found at concentrations below 100 ng/item, demonstrating the ability of the methodology to discriminate differences at concentrations approximating to an order of magnitude lower than the applied 100 ng/item threshold. Four additional compounds, not present within any list of priority toxicants currently used for the regulatory reporting of tobacco products, were subsequently highlighted to be of toxicological concern, namely glycidol, 3-monochloropropane-1,2-diol (3-MCPD), 2-furanmethanol, and furfural [29]. In response to these findings, presented to the FDA in May 2017, as part of PMP’s Premarket Tobacco Product Application (PMTA) for the marketing of *IQOS*® in the USA, the FDA summarized their review of these toxicological findings with the statement that “Although some of the chemicals are genotoxic or cytotoxic, these chemicals are present in very low levels and potential effects are outweighed by the substantial decrease in the number and levels of HPHCs found in combusted cigarettes” [30]. It is clear that the priority toxicants currently selected for regulatory reporting of tobacco products by different authorities (e.g., FDA reduced list of 18 HPHCs for the USA [15], or Health Canada list of 44 HPHCs for Canada [15]) remain applicable for the assessment of heated tobacco products. For toxicants that have been observed to be increased in, or specific to, heated tobacco products compared with combusted cigarettes, their inclusion in specific lists of HPHCs for such products could be considered. However, the small increase in number for such specific HPHCs should be strongly weighed and evaluated in the context of the overall reduction in the numbers and quantities of chemical constituents, including known HPHCs [17], and additionally assessed using biological assays that evaluate the aerosol as a whole.

To the best of our knowledge, this is the first time that such a comprehensive in-depth chemical characterization of the aerosol composition of a heat-not burn product has been reported. This work represents several years of effort in the field of analytical method development and advanced structural identification techniques, which have been applied to the aerosol of THS2.2.

## Electronic supplementary material

ESM 1 (PDF 1349 kb)
