# Supplementary material for: Comprehensive chemical characterization of the aerosol generated by a heated tobacco product by untargeted screening
Source: Anal Bioanal Chem. 2020 Feb 18;412(11):2675–85. doi: 10.1007/s00216-020-02502-1 (PMC7136312; doi:10.1007/s00216-020-02502-1)
Supplement: Supplementary file 1 — (PDF 1349 kb) [file 216_2020_2502_MOESM1_ESM.pdf]

Analytical and Bioanalytical Chemistry

Electronic Supplementary Material

Comprehensive chemical characterization of the aerosol generated by a heated tobacco product by untargeted screening

Mark C. Bentley, Martin Almstetter, Daniel Arndt, Arno Knorr, Elyette Martin,
Pavel Pospisil, Serge Maeder

SAMPLE COLLECTION AND ANALYSIS

Research Materials

The commercialized Tobacco Heating System (THS) 2.2 test items were produced at Philip Morris Manufacturing & Technology Bologna (PMMTB), Italy. The samples were stored in a climatic chamber in packs at 22 ± 3°C and 60 ± 5% relative humidity (RH) prior to conditioning and aerosol collection. The reference cigarette 3R4F was purchased from the University of Kentucky, Kentucky Tobacco Research and Development Center, and stored in a cooling chamber at 4 ± 3°C with uncontrolled humidity prior to conditioning and aerosol collection.

Prior to aerosol generation, the test items were conditioned in accordance with ISO 3402 (ISO 3402 "Tobacco and tobacco products -- Atmosphere for conditioning and testing 4th edition" [International Standard], available from: https://www.iso.org/obp/ui/#iso:std:iso:3402:ed-4:v1:en) for a minimum of 48 h and maximum of 10 days at 22 ± 1°C and 60 ± 3% RH. All test items were conditioned in open packages.

Aerosol Generation and Sample Collection

LC-HRAM-MS Analysis

The list of experimental groups defined and used for LC-HRAM-MS investigations was as follows:

Study Sample Groups for LC-HRAM-MS

Description	Smoking Regimen	Sample Trapping	Type	Short Name
THS 2.2	Health Canada	TPM	Test	THS
3R4F	Health Canada	TPM	Reference	3R4F
Blank	Health Canada	TPM	Reference	Blank
THS 2.2	Health Canada	GVP	Test	THS
3R4F	Health Canada	GVP	Reference	3R4F
Blank THS 2.2	Health Canada	GVP	Reference	BLKT
Blank 3R4F	Health Canada	GVP	Reference	BLK3

Aerosol was generated by using a linear smoking machine and then trapped and treated as required for each of the four LC-HRAM-MS methods (RP-HESI+; RP-HESI-; RP-APCI+, and HILIC-HESI+).

Mainstream aerosols of THS2.2 and 3R4F were generated under Health Canada intense smoking conditions for samples requiring reversed phase (RP) and hydrophilic interaction (HILIC) chromatography. The particulate phase (TPM) was trapped by using a 44-mm Cambridge glass fiber filter pad, and gas-vapor phase (GVP) samples were collected downstream from the filter pad by using two consecutive micro-impingers, each containing methanol (10 mL) maintained at approximately -60°C by using a dry ice–isopropanol mixture.

The room conditions for aerosol generation were maintained at 22 ± 2°C and 60 ± 5% RH.

Aerosol Generation for TPM Samples

Short Name	Puff Volume (mL)	Duration (s)	Puff Interval (s)	Puff Count (n)
THS	55	2	30	12
3R4F	55	2	30	10*
Blank	55	2	30	12

*Smoked to a fixed butt length (35 mm), achieved in approximately 10 puffs.

Aerosol Generation for GVP Samples

Short Name	Puff Volume (mL)	Duration (s)	Puff Interval (s)	Puff Count (n)
THS	55	2	30	12
3R4F	55	2	30	10*
BLKT	55	2	30	12
BLK3	55	2	30	10

*Smoked to a fixed butt length (35 mm), achieved in approximately 10 puffs.

For characterization of TPM, each sample replicate comprised the accumulation of particulate matter from two sticks/cigarettes (items), which was then extracted from the Cambridge filter pad by using methanol (2 x 5 mL) for RP analysis or acetonitrile (2 x 5 mL) for HILIC analysis. GVP samples were trapped directly in methanol by using five items per replicate for THS and three items per replicate for 3R4F (HILIC separation was not required for GVP samples). Blank samples were generated by using the same aerosol collection configuration without test items and were processed for analysis in the same way. Internal standards were added to the extracts prior to chromatographic analysis.

Experimental Details for Preparation of Collected Aerosol Samples

Short Name	Sample Type	Replicates (n)	Items Per Replicate (accumulations)	Extraction Solvent	Extraction Volume (mL)	Analysis
THS	TPM	3	2	Methanol	10	RP
3R4F	TPM	3	2	Methanol	10	RP
Blank	TPM	3	-	Methanol	10	RP
THS	TPM	3	2	Acetonitrile	10	HILIC
3R4F	TPM	3	2	Acetonitrile	10	HILIC
Blank	TPM	3	-	Acetonitrile	10	HILIC
THS	GVP	3	5	Methanol	2 x 10	RP
3R4F	GVP	3	3	Methanol	2 x 10	RP
BLKT	GVP	3	-	Methanol	2 x 10	RP
BLK3	GVP	3	-	Methanol	2 x 10	RP

GCxGC-TOFMS Analysis

The list of experimental groups defined and used for GCxGC-TOFMS investigations was as follows:

Study Sample Groups for GCxGC-TOFMS

Description	Smoking Regimen	Sample Trapping	Type	Short Name
THS 2.2	Health Canada	TPM/GVP	Test	THS
Blank_THS	Health Canada	TPM/GVP	Reference	Blank_THS
3R4F	Health Canada	TPM/GVP	Reference	3R4F
Blank_3R4F	Health Canada	TPM/GVP	Reference	Blank_3R4F

Aerosol was generated by using a linear smoking machine and then trapped and treated as required for each of the three GCxGC-TOFMS methods (polar, nonpolar, and volatile).

Mainstream aerosols of THS and 3R4F were generated under Health Canada smoking conditions, and TPM was trapped by using a 44-mm Cambridge glass fiber filter pad. GVP samples were collected downstream from the filter pad by using two consecutive micro-impingers containing either dichloromethane (DCM)/acetone (80:20 v/v; 10 mL) maintained at approximately -80°C or N,N-dimethylformamide (DMF; 10 mL) maintained at approximately -60°C by using a dry ice–isopropanol mixture.

The room conditions for aerosol generation were 22 ± 2°C and 60 ± 5% RH.

Aerosol Generation for TPM/GVP Samples

Short Name	Puff Volume (mL)	Duration (s)	Puff Interval (s)	Puff Count (n)
THSR	55	2	30	12
Blank_THS	55	2	30	12
3R4F	55	2	30	10*
Blank_3R4F	55	2	30	12

*Smoked to a fixed butt length (35 mm), achieved in approximately 10 puffs.

For characterization of TPM, each sample replicate comprised the accumulation of particulate matter from five items for THS and three items for 3R4F, which was then extracted from the Cambridge filter pad by using DCM/acetone (10 mL) for nonpolar/polar compound analysis or DMF (10 mL) for volatile compound analysis. The solvents used for extraction contained internal standards relevant for the methods being performed (non-polar or volatile methods). GVP samples were trapped directly in DCM/acetone (2 x 10 mL) or DMF (2 x 10 mL) by using five items per replicate for THS and three items per replicate for 3R4F. The solvents used for trapping contained internal standards relevant for the methods being performed. The following table lists the internal standards relevant for the individual methods, and indicates their concentration equivalence in relation to THS2.2 samples (5 accumulated items per sample).

Internal Standards for GCxGC-TOFMS Methods

Method	Internal Standard	Conc. [µg/item]	
		GVP	TPM
Non-Polar	4-Aminobiphenyl-d9	4.8	2.4
	Benz(a)pyrene-d12	0.4	0.2
	Decanoic-d19 acid	23.3	11.7
	Isophorone-d8	4.4	2.2
	Isoquinoline-d7	6.0	3.0
	Naphthalene-d8	2.1	1.1
	Phenol-d6	12.6	6.3
	Styrene-d8	4.8	2.4
Polar	Furfural-d4	2.1	2.1
	4-Hydroxy-4-methyl-2-pentanone-d12	5.1	5.1
	5-Hydroxy-2-methyl-d3-pyridine-3,4,6-d3	5.7	5.7
	N-Methylnicotinamide-2,4,5,6-d4	4.0	4.0
	2-Methylbutyric-d9 Acid	10.1	10.1
	2-Methyl-2,4-pentane-d12-diol	8.0	8.0
	Pentanenitrile-d9	3.1	3.1
	Phenol-d6	3.7	3.7
	N-iso-Propyl-d7-acrylamide	2.0	2.0
	Pyridine-d5	1.9	1.9
Volatile	Acetone-d6	10.1	5.1
	Benzene-d6	10.6	5.3
	2-Butanone-d5	9.9	4.9
	Butyraldehyde-d8	7.9	4.0
	Cyclohexene-d10	5.4	2.7
	Cyclopentane-d10	4.8	2.4
	1,2-Dichloroethane-d4	9.0	4.5
	Dimethyl sulfide-d6	2.6	1.3
	Ethyl acetate-d8	3.7	1.8
	Furan-d4	10.9	5.5
	Methacrylonitrile-d5	10.0	5.0
	3-Methylhexane-d16	6.2	3.1
	Propylene oxide-d6	8.4	4.2
	Tetrahydrofuran-d8	5.5	2.7

DCM/acetone samples were subsequently partitioned by adding an equal volume of water. After thorough mixing, followed by centrifugation, the aqueous and organic phases were separated. Internal standards relevant for the polar method were added at this point to the aqueous phase, which was then analyzed by the polar method. The organic phase was dried by using anhydrous sodium sulfate and then submitted for analysis by the nonpolar method. Blank samples were generated by using the same aerosol collection configuration without test items and were processed for analysis in the same way.

Experimental Details for Preparation of Collected Aerosol Samples

Short Name	Methods	Items Per Replicate (accumulations)	Replicates (n)	Extraction Solvent	Extraction Volume (mL)	Sample Type
THS	Nonpolar/Polar	5	3	DCM/Acetone	10	TPM
	Volatile	5	4	N'N-DMF	10	TPM
Blank_THS	Nonpolar/Polar	-	3	DCM/Acetone	10	TPM
	Volatile	-	4	N'N-DMF	10	TPM
3R4F	Nonpolar/Polar	3	3	DCM/Acetone	10	TPM
	Volatile	3	4	N'N-DMF	10	TPM
Blank_3R4F	Nonpolar/Polar	-	3	DCM/Acetone	10	TPM
	Volatile	-	4	N'N-DMF	10	TPM
THS	Nonpolar/Polar	5	3	DCM/Acetone	2 x 10	GVP
	Volatile	5	4	N'N-DMF	2 x 10	GVP
Blank_THS	Nonpolar/Polar	-	3	DCM/Acetone	2 x 10	GVP
	Volatile	-	4	N'N-DMF	2 x 10	GVP
3R4F	Nonpolar/Polar	3	3	DCM/Acetone	2 x 10	GVP
	Volatile	3	4	N'N-DMF	2 x 10	GVP
Blank_3R4F	Nonpolar/Polar	-	3	DCM/Acetone	2 x 10	GVP
	Volatile	-	4	N'N-DMF	2 x 10	GVP

Instruments, Materials and Sample Analysis

LC-HRAM-MS Analysis

LC-HRAM-MS systems comprised the following components:

Mass Spectrometer	Thermo Q Exactive
Autosampler	Thermo Accela PAL
Column Oven	Thermo HotDog 3000
UHPLC Pump	Thermo Accela 1250
Column RP	Thermo Hypersil GOLD (150 x 2.1 mm; 1.9 µm)
Column HILIC	Thermo Accucore HILIC (150 x 2.1 mm; 2.6 µm)

Aliquots (200 µL) of the extracts were diluted with methanol (700 µL) for RP analysis or acetonitrile (700 µL) for HILIC analysis and fortified with stable labeled internal standards prior to analysis. Isophorone-d8 was used as the internal standard for RP HESI+ and RP APCI+ methods, with an added concentration equivalent to 250 µg/item. Decanoic acid-d19 was used as the internal standard for RP HESI-, with an added concentration equivalent to 500 µg/item. For HILIC HESI+ analysis, myosmine-2,4,5,6-d4 was used as the internal standard, with an added concentration equivalent to 125 µg/item. Aliquots (1.5 µL) of the diluted extracts were injected and analyzed by LC-HRAM-MS in full-scan and data-dependent fragmentation modes for compound identification. Each diluted extract was analyzed five times (analytical replicates) in both full-scan and data-dependent fragmentation modes.

To facilitate data processing, a pool sample (mixture of all samples) was prepared for each method and, following analysis, used as a reference for data processing. These pool samples represented the entire chemical space being evaluated.

Samples were analyzed by LC-HRAM-MS by using a Thermo QExactive™ high-resolution mass spectrometer in both full-scan and data-dependent acquisition modes. In total, four different methods were applied in order to cover a wide range of substances with different ionization properties and compound classes. Samples were analyzed by RP chromatography with heated electrospray ionization (HESI) in both positive and negative modes, by atmospheric pressure chemical ionization (APCI) in positive mode, and also by HILIC chromatography with HESI positive ionization. Samples extracted and prepared by using methanol and acetonitrile were analyzed by the RP and HILIC methods, respectively.

The instrument parameters were as follows:

Instrument Scan Events

Scan Event	Scan Event Details	Detection	Fragmentation	Resolution
(1)	Full scan	FTMS	-	70000
(2)	MS/MS top 3 most intense from (1)	FTMS	HCD	17500

Instrument Parameters

Parameter	Setting
**General Parameters**	
In-Source CID [eV]	Off (0.0 eV)
Default Charge State	1
**Full MS**	
Microscans	1
Resolution	70000
AGC Target	3e6
Maximum IT [ms]	100
Scan Range [Da]	80–800
Spectrum Data Type	Profile
**dd-MS^2**	
Microscans	1
Resolution	17500
AGC Target	1e5
Maximum IT [ms]	150
Loop Count	3
TopN	3
Isolation Window [m/z]	4
Scan Range [Da]	80–800
Stepped NCE [eV]	25, 50, 75

Parameter	Setting
**dd Settings**	
Underfill Ratio [%]	1.00
Intensity Threshold	6.7e3
Apex Trigger	Off
Dynamic Exclusion [s]	10

Accurate mass measurements enabled determination of the elemental composition (proposed sum formula) of precursor ions derived from the full-scan analyses as well as the elemental composition of fragments generated by data-dependent fragmentation experiments. In combination, these data provided a high confidence for the correct proposal of compound elemental composition and identification of structural features. Semi-quantification was performed by means of response comparison with stable labeled internal standards of known concentrations.

GCxGC-TOFMS Analysis

GCxGC-TOFMS systems comprised the following components:

Gas chromatograph	Agilent 7890A or equivalent
Mass spectrometer	LECO Pegasus 4D
Autosampler	Agilent 7683 Series
Injector	Agilent 7683B Series
Columns (nonpolar)	1st : DB-5ms, 30-m length, 0.25-mm internal diameter, 0.25-µm thickness
	2nd: DB-17ht, 2.2-m length, 0.10-mm internal diameter, 0.10-µm thickness
Columns (polar)	Pre: SLB-IL60, 2.0-m length, 0.25-mm internal diameter, 0.20-µm thickness
	1st : DB-FFAP, 30-m length, 0.25-mm internal diameter, 0.25-µm thickness
	2nd: VF-624ms, 1.9-m length, 0.15-mm internal diameter, 0.84-µm thickness
Columns (volatile)	1st : DB-624UI, 30-m length, 0.25-mm internal diameter, 1.4-µm thickness
	2nd: DB-FFAP, 2.4-m length, 0.10-mm internal diameter, 0.1-µm thickness

Non-targeted screening by GC×GC-TOFMS comprised three separate analytical methods focused on nonpolar, polar, and highly volatile compounds.

Nonpolar compound analysis:

TPM was collected by using a glass fiber filter pad and extracted with DCM/acetone (80:20 v/v; containing retention index markers and internal standards). GVP was collected separately by using the same solvent mixture. Each extract was partitioned with an equal volume of water, and the organic phase, after drying with anhydrous sodium sulfate, was submitted for GCxGC-TOFMS analysis by using the following conditions:

Instrument	Parameter	Settings
Injector	injector	cool-on-column, track-oven mode
	injection	on-column
	injection volume	0.1 µL
Gas Chromatograph	carrier gas	helium
	flow	1.0 mL/min (constant flow)
	column 1 (1st dimension)	30 m DB-5ms, 0.25 mm ID, 0.25 µm d_f
	column 2 (2nd dimension)	2.2 m DB-17ht, 0.10 mm ID, 0.10 µm d_f
Gas Chromatograph	primary oven temperature program	rate (°C/min) / target temp. (°C) / duration (min)<br>initial / 30.0 / 2.0<br>5.0 / 325.0 / 15.0
	secondary oven temperature program	rate (°C/min) / target temp. (°C) / duration (min)<br>initial / 35.0 / 2.0<br>5.2 / 340.0 / 14.5
Transfer Line	temperature	280°C
Modulator	modulator	enabled
	modulator temperature program	rate (°C/min) / target temp. (°C) / duration (min)<br>initial / 50.0 / 3.0<br>5.0 / 340.0 / 15.0
	2-dimension separation time	6 s
	hot pulse time	1.00 s
	cool time between stages	2.00 s
Mass Spectrometer	acquisition delay	440 s
	mass range	35–700 Da
	data acquisition rate	200 spectra/s
	detector voltage	1450–2000 V
	electron energy	-70 V
	temperature ion source	230°C

Polar compound analysis:

The remaining aqueous layer from the nonpolar sample preparation was used for analysis of polar compounds. Internal standards and retention index marker compounds were added to the aqueous extract prior to direct GC×GC-TOFMS analysis by using the following conditions:

Instrument	Parameter	Settings		
Injector	injector	cool-on-column, track-oven mode		
	injection	on-column		
	injection volume	0.1 µL		
Gas Chromatograph	carrier gas	helium		
	flow	1.0 mL/min (constant flow)		
	pre-column	2 m SLB-IL60, 0.25 mm ID, 0.20 µm d_f		
	column 1 (1st dimension)	30 m DB-FFAP, 0.25 mm ID, 0.25 µm d_f		
	column 2 (2nd dimension)	1.9 m VF-624ms, 0.15 mm ID, 0.84 µm d_f		
	primary oven temperature program	rate (°C/min)	target temp. ((°C)	duration (min)
		initial	35.0	2.0
		5.0	250.0	23.0
	secondary oven temperature program	rate (°C/min)	target temp. (°C)	duration (min)
		initial	55.0	2.0
		4.6	285.0	16.0
Transfer Line	temperature	280°C		
Modulator	modulator	enabled		
	modulator temperature program	rate (°C/min)	target temp. (°C)	duration (min)
		initial	65.0	2.0
		5.0	300.0	19.0
	2-dimension separation time	6 s		
	hot pulse time	1.00 s		
	cool time between stages	2.00 s		
Mass Spectrometer	acquisition delay	450 s		
	mass range	29–700 Da		
	data acquisition rate	200 spectra/s		
	detector voltage	1450–2000 V		
	electron energy	-70 V		
	temperature ion source	230°C		

Volatile compound analysis:

TPM was collected by using a glass fiber filter pad and extracted with DMF (containing retention index markers and internal standards). GVP was collected separately by using the same solvent mixture. Each extract was submitted for GCxGC-TOFMS analysis by using the following conditions:

Instrument	Parameter	Settings		
Injector	injector	cool-on-column, track-oven mode		
	injection	on-column		
	injection volume	0.1 µL		
Gas Chromatograph	carrier gas	helium		
	flow	2.2 mL/min (68 min), 1.2 mL/min (12 min) (ramped flow)		
	column 1 (1st dimension)	30 m DB-624UI, 0.25 mm ID, 1.4 µm d_f		
	column 2 (2nd dimension)	2.4 m DB-FFAP, 0.10 mm ID, 0.1 µm d_f		
	primary oven temperature program	rate (°C/min)	target temp. (°C)	duration (min)
		initial	-20.0	1.0
		5.0	-5.0	0.0
		1.0	50.0	0.0
		5.0	95.0	0.0
		45.0	230.0	9.0
	secondary oven temperature program	rate (°C/min)	target temp. (°C)	duration (min)
		initial	0.0	4.0
		1.0	55.0	0.0
		5.0	100.0	0.0
		45.0	235.0	9.0
Transfer Line	temperature	250°C		
Modulator	modulator	enabled		
	modulator temperature offset to secondary oven	15°C		
	2-dimension separation time	6 s		
Modulator	hot pulse time	1.00 s		
	cool time between stages	2.00 s		
Mass spectrometer	acquisition delay	0 s		
	mass range	29–500 Da		
	data acquisition rate	200 spectra/s		
	detector voltage	1450–2000 V		
	electron energy	-70 V		
	temperature ion source	230°C		

COMPOUNDS AND CONCENTRATION ESTIMATES – FULL LIST

Table S1 The Most Abundant (≥100 ng/item) Chemical Constituents Present in the Aerosol of THS2.2 and Their Corresponding Concentrations in the Smoke of the 3R4F Reference Cigarette

Compound Name	CAS Number	Identification Confidence	Aerosol Fraction	Conc. in THS2.2 (µg/item)	Conc. in 3R4F (µg/item)
1-Hydroxy-2-propanone/1,2-Propenediol°	116-09-6/7333-03-1	Confirmed	TPM	1135	502
Acetic acid	64-19-7	Confirmed	TPM	994°°	2659
Propylene glycol	57-55-6	Confirmed	TPM	643	89.6
1-Monoacetin	106-61-6	Confirmed	TPM	409	434
Acetaldehyde	75-07-0	Confirmed	TPM/GVP	313	1253
Methanol	67-56-1	Confirmed	TPM/GVP	211	361
Solanesol	13190-97-1	Confirmed	TPM	179	3382
Isobutyraldehyde	78-84-2	Confirmed	GVP	116	259
Triacetin	102-76-1	Confirmed	TPM	112	194
Palmitic acid	57-10-3	Confirmed	TPM	105	266
3-(2-Hydroxymethoxy)-propane-1,2-diol	14641-24-8	Confirmed	TPM	100	267
Cembranoid degradation products (18 compounds)#	-	Confirmed	TPM	93.2	193
Isovaleraldehyde	590-86-3	Confirmed	TPM/GVP	88.7	245
13,14-Dihydroretinol	115797-14-3	Confirmed	TPM	79.1	152
Linolenic acid	463-40-1	Confirmed	TPM	57.9	157
Propanal	123-38-6	Confirmed	GVP	57.4	386
2-Methylbutyraldehyde	96-17-3	Confirmed	GVP	54.7	179
Propanoic acid	79-09-4	Confirmed	TPM	53.2	141
3-Pyridinol	109-00-2	Confirmed	TPM	52.8	218
β-Nicotyrine	487-19-4	Confirmed	TPM	52.4	100
Pyranone	28564-83-2	Confirmed	TPM	51.4	44.5
Oleic acid	112-80-1	Confirmed	TPM	50.2	107
Furfural	98-01-1	Confirmed	TPM/GVP	47.4	38.3
2-Monoacetin	100-78-7	Confirmed	TPM	46.8	30.0
Linoleic acid	60-33-3	Confirmed	TPM	43.0	123
2-Furanmethanol	98-00-0	Confirmed	TPM/GVP	37.5	9.47
Acetone	67-64-1	Confirmed	GVP	34.7	268
2,3-Butanedione	431-03-8	Confirmed	TPM/GVP	34.0	127
Anhydro sugar derivative	-	High	TPM	30.8	43.1
Octadecanoic acid	57-11-4	Confirmed	TPM	29.4	72.7
2-Methylfuran	534-22-5	Confirmed	GVP	28.2	175
Furan	110-00-9	Confirmed	GVP	24.3	214
Neophytadiene	504-96-1	Confirmed	TPM	23.8	43.0
1-Linolenoylglycerol	18465-99-1	Confirmed	TPM	23.5	42.8
5-Hydroxymethylfurfural	67-47-0	Confirmed	TPM	23.0	82.1
α-Levantenolide	30987-48-5	Medium	TPM	22.8	74.8
2-Methyl-2-propenal	78-85-3	Confirmed	TPM/GVP	22.0	115
Pentadecanoic acid	1002-84-2	Confirmed	TPM	18.8	32.7

°Semi-quantified concentration represents the sum of concentrations of two tautomers, which interconvert inconsistently during analysis.
°°Concentration determined quantitatively.
#Degradation experiments with suspected precursors confirmed the compound class proposal.
(CAS, Chemical Abstracts Service; THS, tobacco heating system; TPM, total particulate matter; GVP, gas-vapor phase)

Table S1 (continued). The Most Abundant (≥100 ng/item) Chemical Constituents Present in the Aerosol of THS2.2 and Their Corresponding Concentrations in the Smoke of the 3R4F Reference Cigarette

Compound Name	CAS Number	Identification Confidence	Aerosol Fraction	Conc. in THS2.2 (µg/item)	Conc. in 3R4F (µg/item)
3-Chloro-1,2-propanediol	96-24-2	Confirmed	TPM	16.1	8.21
4,6-Dihydroxy-20-nor-2,7-cembradien-12-one	119613-98-8†	High	TPM	14.6	17.8
3-Methylpentanoic acid	105-43-1	Confirmed	TPM	14.5	12.8
5-Methylfurfural	620-02-0	Confirmed	TPM/GVP	14.2	5.25
1H-Pyrrole	109-97-7	Confirmed	TPM/GVP	14.0	24.8
Phytoene	540-04-5	Medium	TPM	13.8	247
Pyridine	110-86-1	Confirmed	TPM/GVP	13.7	68.4
6,10,14,18,22,26-Hexamethyl-5,9,13,17,21,25-heptacosahexaen-2-one	32304-17-9†	Medium	TPM	13.2	75.3
Butanoic acid	107-92-6	Confirmed	TPM	12.7	22.4
1-Acetyloxy-2-propanone	592-20-1	Confirmed	TPM/GVP	12.2	9.23
N-Octanoylnornicotine	38854-10-3	Confirmed	TPM	12.1	100
5,6-Dihydropyridin-2(1H)-one	6052-73-9	Confirmed	TPM	11.8	38.8
Methanethiol	74-93-1	Confirmed	GVP	11.7	22.9
Chloromethane	74-87-3	Confirmed	GVP	11.1	32.1
Heptacosane	593-49-7	Confirmed	TPM	10.2	8.41
α-Tocopherolquinone	7559-04-8	Confirmed	TPM	10.0	40.5
2-Butanone	78-93-3	Confirmed	GVP	10.0	128
3-Hydroxy-2-butanone	513-86-0	Confirmed	TPM/GVP	9.43	11.2
Arachidic acid	506-30-9	Confirmed	TPM	8.91	35.8
α-Cembratriene-diol	57605-80-8†	High	TPM	8.49	0.393
(9*Z*,12*Z*)-18-Hydroxy-9,12-octadecadienoic acid	4546-59-2	High	TPM	8.47	21.9
2-Cyclopentene-1,4-dione	930-60-9	Confirmed	TPM/GVP	8.40	2.01
2-Cyclopenten-1-one	930-30-3	Confirmed	TPM/GVP	8.20	46.9
2H-Pyran-2-one, tetrahydro-5-hydroxy	33691-73-5	Confirmed	TPM	8.16	4.13
2-Furancarboxylic acid, 3-methyl	4412-96-8	Confirmed	TPM	8.06	18.9
trans-Crotonaldehyde	123-73-9	Confirmed	TPM/GVP	7.87	210
8,11-Epoxy-2,6,12-cembratrien-4-ol	75281-94-6†	Medium	TPM	7.79	19.6
Butanal	123-72-8	Confirmed	GVP	7.79	114
trans-Solanone	54868-48-3	Confirmed	TPM/GVP	7.75	12.7
Palmitoleic acid	373-49-9	Confirmed	TPM	7.46	22.0
Isoraimonol	82458-63-7	High	TPM	7.42	11.4
Scopoletin	92-61-5	Confirmed	TPM	7.21	42.3
Anatabine	581-49-7	Confirmed	TPM	7.15	11.8
Behenic acid	112-85-6	Confirmed	TPM	6.57	40.1
2,3-Pentanedione	600-14-6	Confirmed	GVP	6.43	17.0
Hexadecanoic acid, ethyl ester	628-97-7	Confirmed	TPM	6.43	< 0.100
2,5-Dimethylfuran	625-86-5	Confirmed	GVP	6.38	156
Dimethyldisulfide	624-92-0	Confirmed	GVP	6.34	74.1
2-Methyl-3-pyridinol	1121-25-1	Confirmed	TPM	6.23	41.0
5-Oxo-1-tetradecyl-3-pyrrolidinecarboxylic acid	10054-22-5	Medium	TPM	6.16	10.8
α-Tocopherol	10191-41-0	Confirmed	TPM	5.80	25.4

†CAS number corresponds to one of the isomeric forms of this compound.

Table S1 (continued). The Most Abundant (≥100 ng/item) Chemical Constituents Present in the Aerosol of THS2.2 and Their Corresponding Concentrations in the Smoke of the 3R4F Reference Cigarette

Compound Name	CAS Number	Identification Confidence	Aerosol Fraction	Conc. in THS2.2 (µg/item)	Conc. in 3R4F (µg/item)
1,2-Benzenediol	120-80-9	Confirmed	TPM	5.73	56.5
Hydroquinone	123-31-9	Confirmed	TPM	5.71	80.6
2-Hydroxy-3-oxo-butanal	473-80-3	High	TPM/GVP	5.60	6.58
Andrograpanin	82209-74-3	Confirmed	TPM	5.57	61.7
N-Cyclohexylnicotinamide	10354-56-0	Medium	TPM	5.56	48.3
Lignoceric acid	557-59-5	Confirmed	TPM	5.47	30.1
2(5H)-Furanone	497-23-4	Confirmed	TPM	5.45	2.13
2-Methylbutanoic acid	116-53-0	Confirmed	TPM	5.28	8.08
Isoprene	78-79-5	Confirmed	GVP	5.24	49.1
Acrolein	107-02-8	Confirmed	GVP	5.20	463
3-Methylbutanoic acid	503-74-2	Confirmed	TPM	5.13	9.98
6-Methyl-3-pyridinol	1121-78-4	Confirmed	TPM	5.10	21.3
1,4,7,10-Cyclotetradecatetraene, 1,7,11-trimethyl-4(1-methylethenyl)	101159-07-3	High	TPM	4.93	11.9
Butyrolactone	96-48-0	Confirmed	TPM	4.80	1.08
Myristic acid	544-63-8	Confirmed	TPM	4.62	18.7
Stearidonic acid	20290-75-9	Confirmed	TPM	4.56	21.3
Hentriacontane	630-04-6	Confirmed	TPM	4.54	20.4
Benzene	71-43-2	Confirmed	GVP	4.41	106
Acetamide	60-35-5	Confirmed	TPM	4.30	38.7
3-Methylpalmitic acid	42172-35-0	Medium	TPM	4.27	11.7
3-Methylfuran	930-27-8	Confirmed	GVP	4.26	26.2
2-Cyclohexen-1-one, 2,4,4-trimethyl-3-(1,3-butadienyl)	84696-84-4	High	TPM	4.13	21.6
Harmaline	304-21-2	Confirmed	TPM	4.07	61.2
N-Formylnornicotine	3000-81-5	Confirmed	TPM	3.91	53.6
1,3,5,7,11-Cembrapentaene	420793-93-7†	High	TPM	3.89	8.60
Heptadecanoic acid	506-12-7	Confirmed	TPM	3.86	11.8
Octacosanoic acid	506-48-9	Confirmed	TPM	3.81	29.4
Tricosanoic acid	2433-96-7	Confirmed	TPM	3.80	18.3
Farnesylacetone	762-29-8	Confirmed	TPM	3.80	8.82
Toluene	108-88-3	Confirmed	GVP	3.80	77.5
Phenol	108-95-2	Confirmed	TPM	3.74	22.7
N'-Carbomethoxyanabasine	56078-09-2	High	TPM	3.71	53.2
Propanoic acid, 2-oxo-, methyl ester	600-22-6	Confirmed	TPM/GVP	3.70	19.5
2-Methyl-2-butene	513-35-9	Confirmed	GVP	3.60	92.6
1-(1-Oxohexyl)-2-(3-pyridinyl)-pyrrolidine	38854-09-0†	Confirmed	TPM	3.52	34.8
3-[1-(2-Furanylmethyl)-2-pyrrolidinyl]-pyridine	78210-85-2†	Confirmed	TPM	3.52	6.31
Retinol	68-26-8	Confirmed	TPM	3.48	6.82
Shikimic acid	138-59-0	Confirmed	TPM	3.41	23.5
3-Hydroxypalmitic acid	2398-34-7	Confirmed	TPM	3.38	26.9
Sclareolide	1216-84-8	Confirmed	TPM	3.31	4.50
Methylvinylketone	78-94-4	Confirmed	GVP	3.28	74.6

†CAS number corresponds to one of the isomeric forms of this compound.

Table S1 (continued). The Most Abundant (≥100 ng/item) Chemical Constituents Present in the Aerosol of THS2.2 and Their Corresponding Concentrations in the Smoke of the 3R4F Reference Cigarette

Compound Name	CAS Number	Identification Confidence	Aerosol Fraction	Conc. in THS2.2 (µg/item)	Conc. in 3R4F (µg/item)
1-Hydroxy-2-butanone / 1,2-Butenediol°	5077-67-8/50317-11-8	Confirmed	TPM	3.23	4.06
2-Methylpyrazine	109-08-0	Confirmed	TPM/GVP	3.20	27.7
2-Heptadecenoic acid, (2*E*)-	2825-78-7	Confirmed	TPM	3.20	11.9
Norharman	244-63-3	Confirmed	TPM	3.20	43.9
cis-4-Hydroxymethyl-2-methyl-1,3-dioxolane	3674-21-3	Confirmed	TPM	3.18	< 0.100
Nicoteine	366-18-7	Confirmed	TPM	3.18	23.6
10-Nonadecenoic acid	67228-95-9	Medium	TPM	3.16	9.78
2,3-Dihydrofuran	1191-99-7	Confirmed	GVP	3.14	10.1
Cotinine	486-56-6	Confirmed	TPM	3.09	34.6
2-Hydroxy-γ-butyrolactone	19444-84-9	Confirmed	TPM	3.09	13.6
Methylformate	107-31-3	Confirmed	TPM/GVP	3.01	11.5
5-(Hydroxymethyl)dihydro-2(3H)-furanone	10374-51-3	Confirmed	TPM	2.85	18.9
1,4:3,6-Dianhydro-α-D-glucopyranose	4451-30-3	Confirmed	TPM	2.85	77.6
Acetonitrile	75-05-8	Confirmed	GVP	2.85	479
1-(1-Oxobutyl)-2-(3-pyridinyl)-pyrrolidine	69730-91-2†	Medium	TPM	2.81	5.46
2-Pyrrolidinone	616-45-5	Confirmed	TPM	2.81	20.9
2-Hydroxypyridine	72762-00-6	Confirmed	TPM	2.72	39.2
t-Phytol	253686-88-3	Confirmed	TPM	2.70	3.52
5,8,11-Eicosatriynoic acid	13488-22-7	Confirmed	TPM	2.70	4.63
3-Methoxybenzidine	3365-87-5	High	TPM	2.67	29.9
4-Dodecylphenol	104-43-8	Medium	TPM	2.60	7.10
2-Methyltriacontane	1560-72-1	High	TPM	2.56	12.8
Pentacosanoic acid	506-38-7	Confirmed	TPM	2.53	12.0
1-[4-Amino-2-methyl-5-(2-methylphenyl)-1H-pyrrol-3-yl] ethanone	56463-76-4	Medium	TPM	2.49	18.7
Cerotinic acid	506-46-7	Confirmed	TPM	2.48	14.2
Diacetin	102-62-5 or 25395-31-7	Confirmed	TPM	2.47	1.69
1-Keto-α-cyperone	38043-97-9	High	TPM	2.45	8.70
p-Xylene	106-42-3	Confirmed	GVP	2.39	47.8
3-Methylhentriacontane	4981-99-1	High	TPM	2.39	13.0
12-Isopropenyl-1,5,9-trimethyl-2,5,9-cyclotetradecatrien-1-ol	60026-11-1	High	TPM	2.37	30.4
Acrylic acid	79-10-7	Confirmed	TPM	2.36	35.9
2-Methyl-2-cyclopenten-1-one	1120-73-6	Confirmed	TPM/GVP	2.34	35.3
5-Methoxy-3-(2-pyridinylmethyl)-1H-indole	101832-06-8	High	TPM	2.33	33.2
2-Chloro-1,3-propanediol	497-04-1	Confirmed	TPM	2.32	2.30
Vernolic acid	503-07-1	Confirmed	TPM	2.31	14.7
Heneicosanoic acid	2363-71-5	Confirmed	TPM	2.30	9.85
Myosmine	532-12-7	Confirmed	TPM	2.27	20.5
Dihydro-α-ionone	31499-72-6	Confirmed	TPM	2.24	2.89
3,4-Dimethyl-5-pentyl-2-furanundecanoic acid	57818-36-7	Medium	TPM	2.21	8.54
2-Hydroxytetracosanoic acid	544-57-0	Confirmed	TPM	2.19	21.8

°Semi-quantified concentration represents the sum of concentrations of two tautomers, which interconvert inconsistently during analysis.
†CAS number corresponds to one of the isomeric forms of this compound.

Table S1 (continued). The Most Abundant (≥100 ng/item) Chemical Constituents Present in the Aerosol of THS2.2 and Their Corresponding Concentrations in the Smoke of the 3R4F Reference Cigarette

Compound Name	CAS Number	Identification Confidence	Aerosol Fraction	Conc. in THS2.2 (µg/item)	Conc. in 3R4F (µg/item)
Guaiacol	90-05-1	Confirmed	TPM/GVP	2.18	2.33
N-Ethylnorcotinine	359435-41-9	Confirmed	TPM	2.16	46.2
Retinal	116-31-4	Confirmed	TPM	2.13	4.33
3-Oxo-α-ionol	896107-70-3	Confirmed	TPM	2.11	9.64
Stigmasterol	83-48-7	Confirmed	TPM	2.08	21.9
β-Damascenone	23726-93-4	Confirmed	TPM	2.08	9.25
Phenylacetaldehyde	122-78-1	Confirmed	TPM/GVP	2.00	0.733
2-Acetylfuran	1192-62-7	Confirmed	TPM/GVP	1.99	2.38
2,5-Pyrrolidinedione	123-56-8	Confirmed	TPM	1.93	29.2
Triacontanoic acid	506-50-3	Confirmed	TPM	1.92	15.8
Geranylbenzoate	94-48-4	Medium	TPM	1.92	6.60
3-Methyl-pyridine	108-99-6	Confirmed	TPM	1.92	28.4
Furaneol	3658-77-3	Confirmed	TPM	1.92	2.89
2-[2-(4-Nonylphenoxy)ethoxy]ethyldecanoate	-	Medium	TPM	1.90	6.36
1-Butene	106-98-9	Confirmed	GVP	1.89	20.3
Pentacosane	629-99-2	Confirmed	TPM	1.88	1.08
Benzaldehyde	100-52-7	Confirmed	TPM/GVP	1.83	4.50
cis-2,6-Dimethyl-4-piperidinone	13200-35-6	Confirmed	TPM	1.82	1.22
1-(2,3,4,9-Tetrahydro-1H-β-carbolin-1-yl)acetone	69225-88-3	High	TPM	1.80	19.1
3-[1-(5-Ethyl-2-furanyl)-1H-pyrrol-2-yl] pyridine	78210-88-5	Medium	TPM	1.79	32.3
5-Methyl-2-pyridinol	1003-68-5	Confirmed	TPM	1.77	13.1
Nonacosane	630-03-5	Confirmed	TPM	1.77	7.65
3-Methyldotriacontane	20129-49-1	High	TPM	1.74	11.8
Cholest-7-en-3-ol	6036-58-4	Confirmed	TPM	1.71	8.55
(*Z*)-11-Eicosenoic acid	5561-99-9	Confirmed	TPM	1.68	4.07
2,6-Dimethylpyrazine	108-50-9	Confirmed	TPM/GVP	1.68	10.8
1,4-Naphthalenedione, 2,3-dimethyl-6-(4,8,12-trimethyl tridecyl)-	68860-42-4†	Medium	TPM	1.67	9.33
2-Vinylfuran	1487-18-9	Confirmed	GVP	1.62	9.86
Campesterol	474-62-4	Confirmed	TPM	1.58	15.0
cis-2-Butene	590-18-1	Confirmed	GVP	1.53	50.4
1,3-Cyclopentadiene	542-92-7	Confirmed	GVP	1.51	82.8
β-Sitosterol	83-46-5	Confirmed	TPM	1.50	15.2
2-Methyl-2-butenal	497-03-0	Confirmed	GVP	1.49	9.20
2,4-Dimethylcyclopent-4-ene-1,3-dione	65656-90-8	High	TPM/GVP	1.49	2.73
3-Methyl-nonacosane	14167-67-0	High	TPM	1.48	6.39
Ethyl linolenate	1191-41-9	Confirmed	TPM	1.43	0.357
Ricinoleic acid	141-22-0	Confirmed	TPM	1.38	6.59
2-Methyl-3-phenyl-pyrazine	29444-53-9	Confirmed	TPM	1.37	32.8
Dihydrofuranone derivative	-	High	TPM	1.37	73.5
Acrylamide	79-06-1	Confirmed	TPM	1.34	5.81
2-Hydroxy-4,6-dimethylnicotinonitrile	769-28-8	Medium	TPM	1.32	5.92
Glycidol	556-52-5	Confirmed	TPM	1.31	0.439

†CAS number corresponds to one of the isomeric forms of this compound.

Table S1 (continued). The Most Abundant (≥100 ng/item) Chemical Constituents Present in the Aerosol of THS2.2 and Their Corresponding Concentrations in the Smoke of the 3R4F Reference Cigarette

Compound Name	CAS Number	Identification Confidence	Aerosol Fraction	Conc. in THS2.2 (µg/item)	Conc. in 3R4F (µg/item)
3(2H)-Furanone, dihydro-2-methyl-	3188-00-9	Confirmed	TPM/GVP	1.30	0.561
Nicotelline	494-04-2	Confirmed	TPM	1.27	29.5
Pyridine, 3-[1-(5-propyl-2-furanyl)-1H-pyrrol-2-yl]	78210-89-6	Medium	TPM	1.27	30.3
cis-2-Pentene	627-20-3	Confirmed	GVP	1.26	40.0
2-Methyl-1-butene	563-46-2	Confirmed	GVP	1.25	44.9
1-(6-Hydroxy-1-oxooctyl)-2-(3-pyridinyl)-pyrrolidine	77829-17-5	Confirmed	TPM	1.24	38.7
(9*Z*,12*Z*,15*Z*)-18-Hydroxy-9,12,15-octadecatrienoic acid	51327-73-2	High	TPM	1.23	19.4
2,3'-Bipyridine	581-50-0	Confirmed	TPM	1.23	10.5
5-Methylcotinine	1076198-50-9	Confirmed	TPM	1.18	30.0
Heneicosane	629-94-7	Confirmed	TPM	1.17	0.495
cis-2-Methyl-1,3-pentadiene	1501-60-6	Confirmed	GVP	1.17	30.5
4-(3-Hydroxy-2,6,6-trimethyl-1cyclohexen-1-yl)-3-buten-2-one	14398-34-6†	Confirmed	TPM	1.15	4.08
5,6-Dimethyl-3-pyridinol	61893-00-3	Confirmed	TPM	1.15	9.31
Nonadecanoic acid	646-30-0	Confirmed	TPM	1.15	3.90
Styrene	100-42-5	Confirmed	GVP	1.15	22.8
trans-2-Butene	624-64-6	Confirmed	GVP	1.14	30.4
Nicotine-N-oxide	2820-55-5	Confirmed	TPM	1.14	10.8
1H-Pyrrole, 1-methyl-	96-54-8	Confirmed	GVP	1.13	0.762
Methyl-pyroglutamate	4931-66-2	Confirmed	TPM	1.12	12.3
α-Acetylbutyrolactone	517-23-7	Confirmed	TPM	1.11	6.17
Hexadecanoic acid, methyl ester	112-39-0	Confirmed	TPM	1.10	5.67
Nonacosanoic acid	4250-38-8	Confirmed	TPM	1.07	8.85
6-(Heptyloxy)-3-pyridinamine	857219-70-6	High	TPM	1.07	28.7
2-Methyl-3-propyl-5,6-dihydropyrazine	15986-94-4	High	TPM	1.06	4.04
N-Formyl-anatabine	77264-87-0	Confirmed	TPM	1.06	14.0
2-Hydroxy-3-methyl-2-cyclopenten-1-one	80-71-7	Confirmed	TPM	1.06	8.33
2,3-Dimethyl-2-cyclopenten-1-one	1121-05-7	Confirmed	TPM/GVP	1.05	10.9
Ethyl linoleate	544-35-4	Confirmed	TPM	0.988	0.721
Nonanoic acid	112-05-0	Confirmed	TPM	0.968	1.97
Cyclopentanone	120-92-3	Confirmed	GVP	0.954	23.8
5-Hydroxymaltol	1073-96-7	Confirmed	TPM	0.938	0.491
5-Methyl-2(3H)-furanone	591-12-8	Confirmed	TPM/GVP	0.934	2.78
Pentane	109-66-0	Confirmed	GVP	0.922	36.4
cis-13-Docosenoamide	112-84-5	Confirmed	GVP	0.922	2.33
2-Methyldotriacontane	1720-11-2	High	TPM	0.917	6.33
17-Hydroxylinolenic acid	143343-97-9	Medium	TPM	0.905	4.23
Farnesylacetic acid	6040-06-8†	Confirmed	TPM	0.903	4.28
o-Toluic acid	118-90-1	Confirmed	TPM	0.900	14.3
Menthol	1490-04-6	Confirmed	TPM/GVP	0.894	1.16
24-Methylidenelophenol	1176-52-9	High	TPM	0.879	6.80
3-Methyl-2(1H)-pyridinone	1003-56-1	Confirmed	TPM	0.875	7.58
3,22,23-Trihydroxystigmastan-6-one	90524-90-6†	High	TPM	0.875	2.67

† CAS number corresponds to one of the isomeric forms for this compound

Table S1 (continued). The Most Abundant (≥100 ng/item) Chemical Constituents Present in the Aerosol of THS2.2 and Their Corresponding Concentrations in the Smoke of the 3R4F Reference Cigarette

Compound Name	CAS Number	Identification Confidence	Aerosol Fraction	Conc. in THS2.2 (µg/item)	Conc. in 3R4F (µg/item)
Heptacosanoic acid	7138-40-1	Confirmed	TPM	0.874	5.88
Crotonic acid	3724-65-0	Confirmed	TPM	0.866	7.74
trans-4-hydroxymethyl-2-methyl-1,3-dioxolane	3674-22-4	Confirmed	TPM	0.857	< 0.100
cis-4-Methyl-2-pentene	691-38-3	Confirmed	GVP	0.855	37.8
3-Hydroxy-β-damascone	102488-09-5	High	TPM	0.803	3.59
Furancarboxylic acid, methyl ester	611-13-2	Confirmed	TPM/GVP	0.789	0.234
3-Furaldehyde	498-60-2	Confirmed	TPM/GVP	0.789	6.37
trans-3-Penten-2-one	3102-33-8	Confirmed	GVP	0.786	15.6
Loliolide	38274-00-9	Confirmed	TPM	0.782	5.44
α-Cyperone	473-08-5	Confirmed	TPM	0.782	4.35
3-(Furfuryloxy)-1,2-propanediol	20390-21-0	Medium	TPM	0.772	0.912
Vitamin K1	84-80-0	Confirmed	TPM	0.740	4.82
Megastigmatrienone (2 isomers)	5896-02-6	Confirmed	TPM	0.735	3.84
Cholesterol	57-88-5	Confirmed	TPM	0.732	6.90
trans-2-Pentenal	1576-87-0	Confirmed	GVP	0.718	4.75
1,3-Butadiene	106-99-0	Confirmed	GVP	0.716	75.4
3-Methyl-1-butene	563-45-1	Confirmed	GVP	0.706	34.8
Docosane	629-97-0	Confirmed	TPM	0.703	0.509
Dotriacontane	544-85-4	Confirmed	TPM	0.702	4.60
1-Methyl-1,4-cyclohexadiene	4313-57-9	Confirmed	GVP	0.698	7.46
3-Methyl-2-butanone	563-80-4	Confirmed	GVP	0.697	18.2
1-[4-(Dimethylamino)-2-butyn-1-yl]-5-methyl-2-pyrrolidinone	71970-74-6	Medium	TPM	0.686	20.7
4,8,13-Duvatriene-1,3-diol	7220-78-2	Confirmed	TPM	0.685	21.8
Limonene	138-86-3	Confirmed	GVP	0.684	3.49
3-Methyl-2-cyclopenten-1-one	2758-18-1	Confirmed	TPM	0.670	7.14
Thiirane	420-12-2	Confirmed	GVP	0.663	4.37
Cyclo(Pro-Leu)	5654-86-4	Confirmed	TPM	0.652	15.9
2-Ethylfuran	3208-16-0	Confirmed	GVP	0.647	13.3
Acetone cyanohydrin	75-86-5	Confirmed	GVP	0.647	20.2
2(3H)-Furanone, dihydro-5-(1-hydroxyethyl)	27610-27-1	Confirmed	TPM	0.641	0.336
16-Hydroxy-9-hexadecenoic acid	17278-80-7	Confirmed	TPM	0.636	27.1
Pyrrole-2-carboxamide	4551-72-8	Confirmed	TPM	0.624	23.9
5-Methyl-1,3-cyclopentadiene	96-38-8	Confirmed	GVP	0.593	20.9
5-Methyl-2(5H)-furanone	591-11-7	Confirmed	TPM	0.590	0.417
2-Ethylpyrazine	13925-00-3	Confirmed	TPM/GVP	0.586	3.59
2,4-Dimethylfuran	3710-43-8	Confirmed	GVP	0.584	5.97
2-Methyl-butanenitrile	18936-17-9	Confirmed	GVP	0.580	45.9
2-Methyl-5-(prop-1-en-2-yl)-2-vinyltetrahydrofuran	54750-70-8	Confirmed	GVP	0.574	0.128
1-Methyl-3-(1-methylethyl)-2(1H)-pyrazinone	78210-68-1	Medium	TPM	0.570	8.57
Heptane	142-82-5	Confirmed	GVP	0.563	20.0
Phytuberol	56857-64-8	Medium	TPM	0.562	10.2
α-Amylcinnamyl alcohol	101-85-9	Confirmed	TPM	0.558	3.24

Table S1 (continued). The Most Abundant (≥100 ng/item) Chemical Constituents Present in the Aerosol of THS2.2 and Their Corresponding Concentrations in the Smoke of the 3R4F Reference Cigarette

Compound Name	CAS Number	Identification Confidence	Aerosol Fraction	Conc. in THS2.2 (µg/item)	Conc. in 3R4F (µg/item)
3-Hydroxysolavetivone	62623-88-5	High	TPM	0.553	4.22
trans-2-Pentene	646-04-8	Confirmed	GVP	0.552	42.0
6-Ethyl-5,6-dihydro-2H-pyran-2-one	19895-35-3	Confirmed	TPM	0.550	1.74
1-Chloro-2-propanone	78-95-5	Confirmed	GVP	0.546	4.89
Benzylalcohol	100-51-6	Confirmed	TPM	0.544	0.740
Phytone	502-69-2	Confirmed	TPM	0.543	5.70
3-Pyridinebutanol, d-amino	70898-36-1	Medium	TPM	0.541	16.6
Ethylvinylketone	1629-58-9	Confirmed	GVP	0.537	7.46
Triacontane	638-68-6	Confirmed	TPM	0.535	2.80
1-Methyl-1,3-cyclopentadiene	96-39-9	Confirmed	GVP	0.534	26.6
2-Methyl-heptane	592-27-8	Confirmed	GVP	0.529	7.39
Keto-ionone	27185-77-9	Medium	TPM	0.526	4.44
Solerone	29393-32-6	Confirmed	TPM	0.525	1.55
2-Farnesylethanol	67858-77-9†	High	TPM	0.522	10.8
Pentan-2-one	107-87-9	Confirmed	GVP	0.522	40.0
Geranyllinalool	1113-21-9	Confirmed	TPM	0.518	1.90
1-Acetyloxy-2-butanone	1575-57-1	Confirmed	TPM	0.516	0.752
(6E,10E,14E,18E)-2,6,10,15,19,23-Hexamethyl-1,6,10,14,18,22-tetracosahexaen-3-ol	97232-74-1	Medium	TPM	0.505	3.05
6-Methyl-5-hepten-2-one	110-93-0	Confirmed	GVP	0.502	0.742
p-Menthene(Cyclohexene, 1-methyl-4-(1-methylethyl)-)	1195-31-9	Confirmed	GVP	0.492	7.73
1-Pentene	109-67-1	Confirmed	GVP	0.491	44.7
Phenylacetic acid	103-82-2	Medium	TPM	0.489	4.62
2,6,10,14,18-Pentamethyl-2,6,10,14,18-eicosapentaene	75581-03-2†	High	TPM	0.487	10.7
4-Methyl-1-pentene	691-37-2	Confirmed	GVP	0.487	18.0
2-Ethyl-3-pyridinol	61893-02-5	Confirmed	TPM	0.475	4.55
Pyrrolo[1,2-a]pyrazine-1,4-dione, hexahydro-3propyl-	26626-89-1†	Confirmed	TPM	0.474	16.8
3-Methyl-2-butenal	107-86-8	Confirmed	GVP	0.470	1.74
Propanenitrile	107-12-0	Confirmed	GVP	0.465	71.1
Dimethylsulfide	75-18-3	Confirmed	GVP	0.456	1.70
Dihydromaltol	38877-21-3	Confirmed	TPM	0.453	0.341
16-Hydroxy-hexadecanoic acid	506-13-8	Confirmed	TPM	0.447	5.72
3-Hydroxypropionaldehyde	2134-29-4	High	TPM	0.445	0.691
4-Methyl-2-pentanone	108-10-1	Confirmed	GVP	0.445	7.26
2-Methyloctacosane	1560-98-1	High	TPM	0.445	1.81
Methylacrylate	96-33-3	Confirmed	GVP	0.438	3.53
3-Hexene-2,5-dione	4436-75-3	Confirmed	GVP	0.437	3.92
2-Cyclohexen-1-one	930-68-7	Confirmed	TPM/GVP	0.436	1.55
13'-Hydroxy-γ-tocopherol	1215088-63-3	High	TPM	0.430	1.29
Squalene	111-02-4	Confirmed	TPM	0.429	2.54
2-Methyl-propanoic acid	79-31-2	Confirmed	TPM	0.425	2.45
12-Oxo-phytodienoic acid	67204-66-4	Medium	TPM	0.422	2.02

† CAS number corresponds to one of the isomeric forms for this compound

Table S1 (continued). The Most Abundant (≥100 ng/item) Chemical Constituents Present in the Aerosol of THS2.2 and Their Corresponding Concentrations in the Smoke of the 3R4F Reference Cigarette

Compound Name	CAS Number	Identification Confidence	Aerosol Fraction	Conc. in THS2.2 (µg/item)	Conc. in 3R4F (µg/item)
[1-Methyl-3-oxo-2-pentylidenecyclopentyl]acetic acid, (2*E*)-	958790-53-9	High	TPM	0.418	3.97
Tricosanal	72934-02-2	Confirmed	TPM	0.417	1.64
Stigmasta-5,7,22,25-tetraen-3-ol	119386-11-7	High	TPM	0.409	2.84
Higher molecular weight derivative of Farnesylacetone (C22)	-	High	TPM	0.408	2.02
2-Methyl-pyridine	109-06-8	Confirmed	TPM/GVP	0.407	14.3
β-Damascone	23726-91-2	Confirmed	TPM	0.402	1.37
2-Methylpentane	107-83-5	Confirmed	GVP	0.401	12.2
Higher molecular weight derivative of Farnesylacetone (C26)	-	High	TPM	0.401	2.38
2,3-Dimethylfuran	14920-89-9	Confirmed	GVP	0.394	4.89
2-Hydroxycerotic acid	14176-13-7	Medium	TPM	0.387	4.35
1-Ethyl-9H-pyrido[3,4-ß]indole	20127-61-1	Confirmed	TPM	0.378	12.1
2-Methylbutane	78-78-4	Confirmed	GVP	0.378	21.3
Glutarimide	1121-89-7	Confirmed	TPM	0.370	8.24
Plastoquinone 3	1168-52-1	High	TPM	0.365	1.00
3,4-Hexanedione	4437-51-8	Confirmed	GVP	0.362	1.39
2-Hydroxy-2-cyclopenten-1-one / 1,2-Cyclopentanedione°	10493-98-8/3008-40-0	Confirmed	TPM	0.358	0.440
Scoparone	120-08-1	Confirmed	TPM	0.357	2.25
Cyclopentene	142-29-0	Confirmed	GVP	0.355	20.5
Ethylbenzene-	100-41-4	Confirmed	GVP	0.354	26.8
2,3-Dihydro-1,4-dioxin	543-75-9	Confirmed	GVP	0.346	2.32
Dihydro-4-hydroxy-2(3H)-furanone	7331-52-4	Confirmed	TPM	0.340	4.78
Cyclohexene, 3-(2-propenyl)- or Cyclohexene, 3-(1-methylethyl)-	5232-95-8 or 3983-08-2	Medium	GVP	0.333	4.95
trans-1,3-Pentadiene	2004-70-8	Confirmed	GVP	0.330	64.6
Pentan-3-one	96-22-0	Confirmed	GVP	0.328	14.1
Pyrazine	290-37-9	Confirmed	TPM/GVP	0.318	2.04
2-Methyl-2-propenoic acid	79-41-4	Confirmed	TPM	0.311	2.70
Cyclohexylphenylacetic acid	3894-09-5	Confirmed	TPM	0.309	3.68
Pentanal	110-62-3	Confirmed	GVP	0.308	3.08
3-Oxo-7,8-dihydro-a-ionol	60047-19-0	Confirmed	TPM	0.307	1.75
2,3-Dimethylpyrazine	5910-89-4	Confirmed	TPM/GVP	0.307	2.79
Geranylacetone	3796-70-1	Confirmed	TPM	0.294	1.39
3(2H)-Furanone	3511-31-7	Medium	TPM	0.294	< 0.100
2-Oxo-propionamide	631-66-3	Confirmed	TPM	0.291	4.95
trans-2,6-Octadiene, 2,6-dimethyl-	2609-23-6	Confirmed	GVP	0.288	6.93
Glycidylacetate	6387-89-9	Confirmed	TPM	0.282	0.211
Cyclo(Phe-Pro)	3705-26-8	Confirmed	TPM	0.280	10.3
Harman	486-84-0	Confirmed	TPM	0.278	25.5
6,10,14,18-Tetramethyl-5,9,13,17-nonadecatetraen-2-one	6809-52-5	Confirmed	TPM	0.278	0.902
Squalene derivative (C28)	-	Medium	TPM	0.277	7.55
Norsolanadione	60619-46-7	High	TPM	0.275	3.14
2-Cyclopenten-1-one, dimethyl- (configurational isomer 1)	-	High	TPM/GVP	0.273	8.16

° semi-quantified concentration represents the sum of 2 tautomers, which interconvert inconsistently during analysis

Table S1 (continued). The Most Abundant (≥100 ng/item) Chemical Constituents Present in the Aerosol of THS2.2 and Their Corresponding Concentrations in the Smoke of the 3R4F Reference Cigarette

Compound Name	CAS Number	Identification Confidence	Aerosol Fraction	Conc. in THS2.2 (µg/item)	Conc. in 3R4F (µg/item)
Higher molecular weight derivative of Farnesylacetone (C30)	-	High	TPM	0.268	2.13
Norcotinine	5980-06-3	Confirmed	TPM	0.263	6.83
3-Hydroxy-4-methylbenzoic acid	586-30-1	High	TPM	0.260	2.09
1H-Pyrrole, 1-ethyl-	617-92-5	Confirmed	GVP	0.258	0.136
Benzene, 2-(1,3-butadienyl)-1,3,5-trimethyl-	5732-00-3	Confirmed	TPM	0.255	0.472
Isofucosterol	481-14-1	High	TPM	0.255	3.24
Octadecanoic acid, ethyl ester	111-61-5	Confirmed	TPM	0.253	< 0.100
1,2-Propadiene	463-49-0	Confirmed	GVP	0.249	19.5
Steroid derivative	-	High	TPM	0.247	2.06
trans-3-Methyl-2-pentene	616-12-6	Confirmed	GVP	0.246	24.7
Higher molecular weight derivative of α-Longipinene or α-Neoclovene	-	High	TPM	0.242	1.71
4-Ethenyl-2,6-dimethoxy-phenol	28343-22-8	Confirmed	TPM	0.241	5.75
3-Hexanone	589-38-8	Confirmed	GVP	0.240	1.60
N-Acetylanatabine	91565-91-2	Confirmed	TPM	0.239	14.6
p-Cresol	106-44-5	Confirmed	TPM	0.236	20.2
Formic acid	64-18-6	Confirmed	TPM	0.233	12.8
2-Ethyl-1-butene	760-21-4	Confirmed	GVP	0.233	5.11
Adenine	73-24-5	Confirmed	TPM	0.231	5.95
Caprolactone	502-44-3	Confirmed	TPM	0.229	< 0.100
2-Heptanone	110-43-0	Confirmed	GVP	0.229	1.88
Octacosane	630-02-4	Confirmed	TPM	0.228	0.954
2-Acetylpyrrole	1072-83-9	Confirmed	TPM	0.225	0.371
N-Acetylanabasine	3350-86-5	Confirmed	TPM	0.221	7.26
Norfuraneol	19322-27-1	Confirmed	TPM	0.219	0.390
2-Heptanone, 6-methyl-	928-68-7	Confirmed	GVP	0.215	0.679
Benzoic acid, 2-hydroxy-4-methyl	50-85-1	Medium	TPM	0.214	1.74
N-acetyl-4(H)-pyridine	67402-83-9	High	TPM	0.213	< 0.100
Butyl-1H-imidazole	50790-93-7	Confirmed	TPM	0.213	6.18
4-Pentenal	2100-17-6	Confirmed	GVP	0.213	2.95
Phenol, 4-ethenyl-	2628-17-3	Confirmed	TPM	0.206	15.0
Methylpropionate	554-12-1	Confirmed	GVP	0.199	1.32
2,7,11-Trimethyl-1,6,10-dodecatriene	502723-87-7	High	TPM	0.198	6.147
2-Acetyl-2-hydroxy-g-butyrolactone	135366-64-2	Confirmed	TPM	0.197	0.354
Labdanediol	10267-21-7†	High	TPM	0.196	< 0.100
Eicosane	112-95-8	Confirmed	TPM	0.194	0.247
2-Cyclopenten-1-one, 3-ethyl-2-hydroxy-	21835-01-8	Confirmed	TPM	0.194	1.66
Nicotinamide	98-92-0	Confirmed	TPM	0.192	4.32
Dimethyltrisulfide	3658-80-8	Confirmed	GVP	0.192	0.460
Hydrogen sulfide	7783-06-4	Confirmed	GVP	0.190	5.04
2,5-Hexanedione	110-13-4	Confirmed	TPM	0.190	0.515
2-Methyl-3-pentanone	565-69-5	Confirmed	GVP	0.189	1.21
2,3-Dimethyl-1-butene	563-78-0	Confirmed	GVP	0.188	16.9

† CAS number corresponds to one of the isomeric forms for this compound

Table S1 (continued). The Most Abundant (≥100 ng/item) Chemical Constituents Present in the Aerosol of THS2.2 and Their Corresponding Concentrations in the Smoke of the 3R4F Reference Cigarette

Compound Name	CAS Number	Identification Confidence	Aerosol Fraction	Conc. in THS2.2 (µg/item)	Conc. in 3R4F (µg/item)
Pentadecanal	2765-11-9	Confirmed	TPM	0.183	1.49
trans-2-Methyl-1,3-pentadiene	926-54-5	Confirmed	GVP	0.182	19.3
1-(4-Methylphenyl)-ethanone	122-00-9	Confirmed	TPM	0.182	0.754
cis-3-Methyl-2-pentene	922-62-3	Confirmed	GVP	0.179	21.2
3'-Hydroxycotinine	34834-67-8	Confirmed	TPM	0.178	7.22
4-Vinylguaiacol	7786-61-0	Confirmed	TPM	0.177	6.24
2,2,6-Trimethyl-1-(3-methylbuta-1,3-dienyl)-7-oxabicyclo[4.1.0]heptan-3-ol	1427305-74-5	Medium	TPM	0.177	3.48
Maltol	118-71-8	Confirmed	TPM	0.175	0.347
3,4-Dimethyl-2,5-furandione	766-39-2	Confirmed	TPM	0.174	0.235
3-Methyl-2(5H)-furanone	22122-36-7	Confirmed	TPM	0.174	0.295
2H-Pyrrol-2-one, 4-ethyl-1,5-dihydro-3-methyl	766-45-0	High	TPM	0.173	12.4
(2-Isopropyl-1,3-dioxolan-4-yl)methanol	31192-94-6	High	TPM	0.171	< 0.100
α-Ionol	25312-34-9	Confirmed	GVP	0.169	< 0.100
10-Heneicosene	95008-11-0	Confirmed	TPM	0.167	0.442
2-Methylhydroquinone	95-71-6	Confirmed	TPM	0.166	4.86
1-Heptene	592-76-7	Confirmed	GVP	0.165	23.9
Isobutyronitrile	78-82-0	Confirmed	GVP	0.165	20.9
5-Methyl-2-furanmethanol	3857-25-8	Confirmed	TPM	0.164	< 0.100
5-Isopropyl-2,4-imidazolidinedione	16935-34-5	Confirmed	TPM	0.163	8.36
Pantolactone	79-50-5	Confirmed	TPM	0.163	0.278
cis-2,6-Octadiene, 2,6-dimethyl-	2492-22-0	Confirmed	GVP	0.160	4.44
1-Methylcyclopentene	693-89-0	Confirmed	GVP	0.160	19.4
Isocrotonic acid	503-64-0	Confirmed	TPM	0.159	1.53
2,5-Dihydro-3,5-dimethyl-2-furanone	5584-69-0	High	TPM	0.158	0.209
9-Eicosyne	71899-38-2	High	TPM	0.158	0.941
2-Methylphenol	95-48-7	Confirmed	TPM	0.158	6.73
Butyl-hydroxytoluene	128-37-0	Confirmed	TPM	0.155	< 0.100
c-Phytol	854039-21-7	Confirmed	TPM	0.154	0.222
2-Vinyl-5-methylfuran	10504-13-9	Confirmed	GVP	0.153	0.330
trans-4-Methyl-2-pentene	674-76-0	Confirmed	GVP	0.151	7.43
cis-3-Hexene	7642-09-3	Confirmed	GVP	0.150	5.25
Cyclohexene	110-83-8	Confirmed	GVP	0.149	7.85
Diacetin monopropanoate	36600-62-1	Confirmed	TPM	0.149	1.69
1-Docosanol	661-19-8	Confirmed	TPM	0.149	0.534
4-Vinylpyridine	100-43-6	Confirmed	TPM	0.148	12.7
5-Cyanonicotine	42459-12-1	Confirmed	TPM	0.146	59.5
Thiophene	110-02-1	Confirmed	GVP	0.145	1.92
4-Vinylcatechol	6053-02-7	Confirmed	TPM	0.140	38.3
9-Nonadecene	31035-07-1	High	TPM	0.137	2.61
4-Ethylguaiacol	2785-89-9	Confirmed	TPM	0.137	0.377
cis-Verbenol	18881-04-4	Confirmed	TPM	0.137	1.13
1-Hexadecanol	36653-82-4	Confirmed	TPM	0.136	0.833
2-Cyclopenten-1-one, dimethyl- (configurational isomer 2)	-	High	GVP	0.135	3.02

Table S1 (continued). The Most Abundant (≥100 ng/item) Chemical Constituents Present in the Aerosol of THS2.2 and Their Corresponding Concentrations in the Smoke of the 3R4F Reference Cigarette

Compound Name	CAS Number	Identification Confidence	Aerosol Fraction	Conc. in THS2.2 (µg/item)	Conc. in 3R4F (µg/item)
trans-Caryophyllene	87-44-5	Confirmed	TPM	0.135	1.22
7-Oxabicyclo[4.1.0]heptan-3-ol, 6-(3-hydroxy-1-butenyl)-1,5,5-trimethyl-)-	72777-88-9	Medium	TPM	0.135	1.31
1-Methylcyclohexene	591-49-1	Confirmed	GVP	0.134	2.78
Phenylethyl alcohol	60-12-8	Confirmed	TPM	0.133	0.381
Chloroethane	75-00-3	Confirmed	GVP	0.131	1.06
Cyclobutanone	1191-95-3	Confirmed	GVP	0.130	3.79
Linolenic acid, methyl ester	301-00-8	Confirmed	TPM	0.130	0.809
trans-2-Hexene	13269-52-8	Confirmed	GVP	0.130	17.4
Butanenitrile, 2-methylene	1647-11-6	Confirmed	GVP	0.130	12.3
2,4-Dimethyl pyridine	108-47-4	Confirmed	TPM	0.129	4.35
3-Acetoxypyridine	17747-43-2	Confirmed	TPM	0.128	8.17
Tridecane, 2,6,10-trimethyl-	3891-99-4	High	TPM	0.126	1.67
2-Methyl-2-hexene	2738-19-4	Confirmed	GVP	0.126	17.6
3,5-Dimethylcyclopentene	7459-71-4	High	GVP	0.126	7.18
2-Methylcyclopentanone	1120-72-5	Confirmed	GVP	0.125	6.85
3-(4,8,12-Trimethyltridecyl)furan	54869-11-3	Confirmed	TPM	0.125	0.260
N-Nitrosoanatabine	887407-16-1	Confirmed	TPM	0.125	0.955
4-Ethylcatechol	1124-39-6	Confirmed	TPM	0.124	5.84
Hexacosane	630-01-3	Confirmed	TPM	0.123	0.360
Bulnesol	22451-73-6	High	TPM	0.122	< 0.100
Nonane	111-84-2	Confirmed	GVP	0.121	3.34
Eicosane, 2-methyl-	1560-84-5	Confirmed	TPM	0.121	< 0.100
2-Furancarbonitrile	617-90-3	Confirmed	GVP	0.120	1.67
2-Methyl-1,4-pentadiene	763-30-4	Confirmed	GVP	0.119	9.84
1,2-Cyclohexanedione	765-87-7	Confirmed	TPM	0.119	< 0.100
trans,trans-2,4-Hexadiene	5194-51-4	Confirmed	GVP	0.119	16.3
2-Tritriacontanone	75207-55-5	High	TPM	0.118	2.00
1,3-Cyclohexadiene	592-57-4	Confirmed	GVP	0.115	10.3
Dococosanal	57402-36-5	Confirmed	TPM	0.114	0.315
Sinapyl alcohol	537-33-7	Confirmed	TPM	0.114	7.95
Squalene oxide	7200-26-2	Confirmed	TPM	0.113	1.49
Octane, 3,3-dimethyl-	4110-44-5	Confirmed	GVP	0.111	0.610
N-Furfurylpyrrole	1438-94-4	Confirmed	TPM	0.108	0.103
3-Methyltritriacontane	14167-69-2	High	TPM	0.108	0.853
Octane	111-65-9	Confirmed	GVP	0.108	6.73
o-Cymene	527-84-4	Confirmed	GVP	0.106	0.323
9-Methyladenine	700-00-5	Confirmed	TPM	0.106	1.02
2-Methylnonadecane	1560-86-7	Confirmed	TPM	0.106	< 0.100
2(3H)-Furanone, dihydro-3-hydroxy-4,4-dimethyl-, (R)-	599-04-2	Confirmed	TPM	0.105	0.202
Nonadecane	629-92-5	Confirmed	TPM	0.105	0.194
2-Ethylpyridine	100-71-0	Confirmed	GVP	0.101	0.276
3-Methylcinnamic acid	3029-79-6	Medium	TPM	0.101	4.26
2,5-Dimethyl-3(2H)-furanone	14400-67-0	Confirmed	TPM	0.100	< 0.100
Not Identified (GCxGC-TOFMS; 10 compounds)	-	Not Identified	TPM	7.33	8.38
Not Identified (GCxGC-TOFMS; 3 compounds)	-	Not Identified	GVP	5.18	3.70
